# Drugging an undruggable pocket on KRAS

**DOI:** 10.1073/pnas.1904529116

**Published:** 2019-07-22

**Authors:** Dirk Kessler, Michael Gmachl, Andreas Mantoulidis, Laetitia J. Martin, Andreas Zoephel, Moriz Mayer, Andreas Gollner, David Covini, Silke Fischer, Thomas Gerstberger, Teresa Gmaschitz, Craig Goodwin, Peter Greb, Daniela Häring, Wolfgang Hela, Johann Hoffmann, Jale Karolyi-Oezguer, Petr Knesl, Stefan Kornigg, Manfred Koegl, Roland Kousek, Lyne Lamarre, Franziska Moser, Silvia Munico-Martinez, Christoph Peinsipp, Jason Phan, Jörg Rinnenthal, Jiqing Sai, Christian Salamon, Yvonne Scherbantin, Katharina Schipany, Renate Schnitzer, Andreas Schrenk, Bernadette Sharps, Gabriella Siszler, Qi Sun, Alex Waterson, Bernhard Wolkerstorfer, Markus Zeeb, Mark Pearson, Stephen W. Fesik, Darryl B. McConnell

**Affiliations:** ^a^Discovery Research, Boehringer Ingelheim Regional Center Vienna GmbH & Co KG, 1120 Vienna, Austria;; ^b^Department of Biochemistry, Vanderbilt University School of Medicine, Nashville, TN 37235;; ^c^Discovery Research, Boehringer Ingelheim Pharma GmbH & Co KG, D-88397 Biberach an der Riss, Germany;; ^d^Department of Pharmacology, Vanderbilt University School of Medicine, Nashville, TN 37235;; ^e^Department of Chemistry, Vanderbilt University, Nashville, TN 37235

**Keywords:** KRAS, NMR, oncology, structure-based drug design, fragment-based drug design

## Abstract

The 3 human RAS genes, KRAS, NRAS, and HRAS, encode 4 different RAS proteins which belong to the protein family of small GTPases that function as binary molecular switches involved in cell signaling. Activating mutations in RAS are among the most common oncogenic drivers in human cancers, with KRAS being the most frequently mutated oncogene. Although KRAS is an excellent drug discovery target for many cancers, and despite decades of research, no therapeutic agent directly targeting RAS has been clinically approved. Using structure-based drug design, we have discovered BI-2852 (1), a KRAS inhibitor that binds with nanomolar affinity to a pocket, thus far perceived to be “undruggable,” between switch I and II on RAS; 1 is mechanistically distinct from covalent KRAS^G12C^ inhibitors because it binds to a different pocket present in both the active and inactive forms of KRAS. In doing so, it blocks all GEF, GAP, and effector interactions with KRAS, leading to inhibition of downstream signaling and an antiproliferative effect in the low micromolar range in KRAS mutant cells. These findings clearly demonstrate that this so-called switch I/II pocket is indeed druggable and provide the scientific community with a chemical probe that simultaneously targets the active and inactive forms of KRAS.

## Significance

Despite decades of research, no approved drugs have been discovered for KRAS. Recently, a pocket occurring on the surface of the active and inactive form of KRAS was found, but, due to its comparatively shallow, polar nature, this pocket has been assumed to be “undruggable.” Starting from very weakly binding fragments and using structure-based drug design, we discovered BI-2852 (1), a nanomolar inhibitor to this pocket which is mechanistically distinct to covalent KRAS^G12C^ inhibitors; 1 modulates pERK and pAKT and has an antiproliferative effect in KRAS mutant cells. This work demonstrates the druggability of this so-called switch I/II pocket and provides the scientific community with a chemical probe that directly inhibits the active and inactive forms of KRAS.

The 3 human *RAS* genes, *KRAS*, *NRAS*, and *HRAS,* encode 4 different RAS proteins (KRAS-4A, KRAS-4B, NRAS, and HRAS) which belong to the protein family of small GTPases that function as binary molecular switches involved in cell signaling (1). Activating mutations in *RAS* like the glycine 12 mutations are among the most common oncogenic drivers in human cancers. *KRAS* is the most frequently mutated oncogene, with mutation rates of 86 to 96% in pancreatic cancers (2), 40 to 54% in colorectal cancers (3), and 27 to 39% in lung adenocarcinomas (4). *NRAS* is predominantly mutated in melanoma and hematological malignancies (5, 6), while HRAS mutations are found in salivary gland and urinary tract cancers (7, 8).

The RAS family is known to cycle through 2 different conformational states that are defined by differential binding to nucleotides. In the “off” state, RAS proteins are bound to the nucleotide guanosine diphosphate (GDP), while in the “on” state they are bound to the nucleotide guanosine triphosphate (GTP). The γ-phosphate of GTP holds 2 regions, switch I and switch II (9), in a compact conformation that allows interaction with downstream effectors, such as CRAF, PI3Kα, and RALGDS, as well as with the allosteric site of SOS1 and SOS2. Hydrolysis of the γ-phosphate to produce GDP-RAS causes a conformational change in the switch regions, leading to the formation of an inactive state which is unable to bind effector molecules (10, 11). RAS itself has an intrinsic, but weak, GTPase activity that is enhanced by GTPase-activating proteins (GAPs) catalyzing RAS inactivation. The exchange of the bound nucleotide GDP into GTP is facilitated by guanine nucleotide exchange factors (GEFs) which, in the case of KRAS, is performed by SOS1 and SOS2 (12). GEFs catalyze the release of GDP from RAS in the cytoplasm and replace it with the more abundant intracellular GTP. Oncogenic mutations in RAS impair GTP hydrolysis, leading to stabilization of the activated GTP-RAS form and enhanced RAS signaling. The most common mutations occur as single-point mutations at codons 12, 13, and 61 (13).

Although KRAS could serve as an excellent drug target for many cancers, direct inhibition of oncogenic RAS has proven to be challenging. Despite decades of research, no therapeutic agent directly targeting RAS has been clinically approved. The main reason for this is the lack of druggable pockets on the surface of RAS. However, in recent years, there has been a resurgence of research around RAS, driven by the growing belief that RAS might be able to be drugged with low molecular weight organic molecules. This belief was sparked by the discovery of 2 pockets on the surface of RAS that could potentially be amenable to small-molecule drug discovery. The S.W.F. group at Vanderbilt (14), researchers at Genentech (15), and, more recently, the Rabbitts group (16, 17) discovered small molecules that bind to a shallow pocket between the switch I and II regions of KRAS. This pocket will be referred to as the switch I/II pocket (SI/II-pocket). In addition, the Shokat group discovered covalently linked small molecules which bind to a second pocket on RAS positioned above the switch II loop in GDP-KRAS^G12C^, called the switch II pocket (SII-pocket) (11).

In this paper, we describe the discovery of nanomolar inhibitors that directly target the small, polar SI/II-pocket present on both the active and inactive form of KRAS. To discover small molecules that bind to KRAS, we conducted several fragment-based screens using uniformly ^15^N-labeled guanosine-5′-[(β,γ)-methyleno]triphosphate (GCP)-bound KRAS^G12D^ for validation. From these screens, we identified fragments that weakly bind to GCP-KRAS^G12D^ that were optimized using structure-based design. This was accomplished by developing a robust system for crystallizing small molecules bound to GTP-KRAS^G12D^. The most potent KRAS inhibitor, **BI-2852 (**1**)**, binds with nanomolar affinity to the active and inactive form of KRAS. Compound **1** blocks the interaction between GDP-KRAS and the catalytic site of SOS1, but, in contrast to covalent KRAS^G12C^ inhibitors, also inhibits the interactions between GTP-KRAS and the allosteric site of SOS1 as well as its effectors (CRAF and PI3Kα). In cells, **1** inhibits SOS1-catalyzed exchange of GDP to GTP as well as GAP-catalyzed exchange of GTP to GDP, which results in no net change in cellular GTP-RAS levels upon treatment. Compound **1** reduced pERK and pAKT levels in a dose-dependent manner, leading to an antiproliferative effect in NCI-H358 cells. The effects of **1** were confirmed to be KRAS-driven and not unspecific effects, through the consistent data generated for the 10-fold weaker distomer **44**. Compound **1** demonstrates that the SI/II-pocket is indeed druggable and provides an ideal starting point for the design of more potent and selective RAS inhibitors. Compound **1** will also serve as a useful chemical probe for the scientific community in the study of RAS biology of simultaneous inhibition of active and inactive RAS in an in vitro setting.

## Results

### GTP-KRAS Fragment Screening.

We adopted multiple approaches to identify inhibitors of KRAS. Initial attempts to find starting points for GTP-KRAS using high-throughput screening of 1.7 million compounds with a luminescent oxygen channeling immunoassay (18), as well as a mammalian protein−protein interaction (PPI) trap cellular assay (19, 20), failed to deliver any hits which could be validated to bind to KRAS in a dose-dependent manner.

**Fig. 1.**
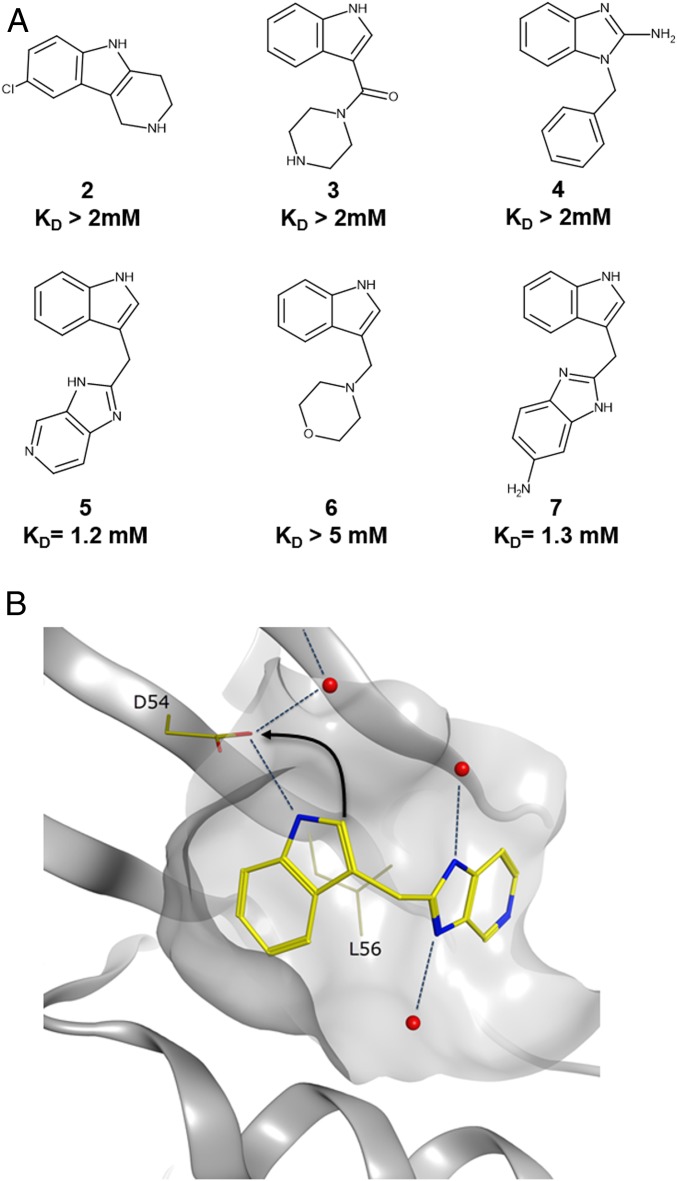
Fragments identified from 2 separate fragment screens. (*A*) Representative indole and benzimidazole fragments identified from the fragment screens. (*B*) The binding mode of indole **5** in GDP-KRAS (Protein Data Bank [PDB] ID code 4EPV) showing the H bond between the indole NH and the side chain of D54. Indole 5 shown in yellow, water molecules shown in red. Arrow indicates the strategy of forming an additional charge−charge interaction with the side chain of D54 from the indole 2 position.

Next, we attempted to identify compounds that bind to GTP-KRAS via fragment-based screening (21–23). A library of 1,800 fragments was screened using both saturation transfer difference NMR (24, 25) and microscale thermophoresis (26) using KRAS^G12V^-phosphomethylphosphonic acid guanylate ester (GCP-KRAS^G12V^), from which 16 fragments were found and subsequently validated (hit rate 0.9%) by the observation of cross-peak shifts in the 2D ^1^H/^15^N heteronuclear single-quantum correlation (HSQC) NMR spectra of GCP-KRAS^G12D^. We also screened a library of 13,800 compounds using uniformly ^15^N-labeled GNP-KRAS by HSQC NMR experiments. Representatives of some of the fragments which bound the active form of KRAS from the 2 screens are depicted in Fig. 1*A*. Despite the large number of hits obtained from the screens (55 in total), the binding affinity of the fragments identified all displayed dissociation constants (K_D_) of greater than 1 mM as measured via HSQC NMR (*SI Appendix*, Fig. S1).

Following the fragment screen, we conducted a follow-up screen of commercially and internally available compounds with high structural similarity to the initial fragment hits, often called “structure activity relationship (SAR) by catalogue” (27, 28). We biased the follow-up SAR by a catalog screen with available indoles bearing a pendent group containing a basic amine at the 2 position, as we hypothesized that forming a charged interaction with D54 in addition to the existing hydrogen bond (H bond) formed by the indole NH (Fig. 1*B*) would lead to a significant improvement in binding affinity. Indeed, indoles containing a methylamino functionality at the 2 position showed a high propensity to bind to GCP-KRAS with measureable K_D_s in the range of 1 mM (*SI Appendix*, Table S1). However, our GCP-KRAS cocrystallization efforts were unsuccessful using any of these more potent fragments from the SAR by catalog screen.

### Further Optimization Leading to Cocrystal Structures.

Although protein NMR is the only biophysical method capable of robustly measuring K_D_s in the millimolar range, the approach is protein-intensive. To facilitate this, a reliable and high-yielding procedure (∼10 mg/L purified yield) for obtaining large amounts of uniformly ^15^N-labeled GCP-KRAS^G12D^ was developed. This was achieved by first expressing the GDP-KRAS from *Escherichia coli*, hydrolyzing GDP to guanidine, and then performing the exchange with the stable GTP analog phosphomethylphosphonic acid guanylate ester (GCP). With this modified approach, we could achieve better yields of uniformly nucleotide-exchanged KRAS compared with the previously described methods using the less stable GTP gamma S or GMPPNP (29). In addition, we used an artificial KRAS T35S mutant for the first optimization steps, as this point mutant was described to display better in HSQC NMR spectra (30).

**Fig. 2.**
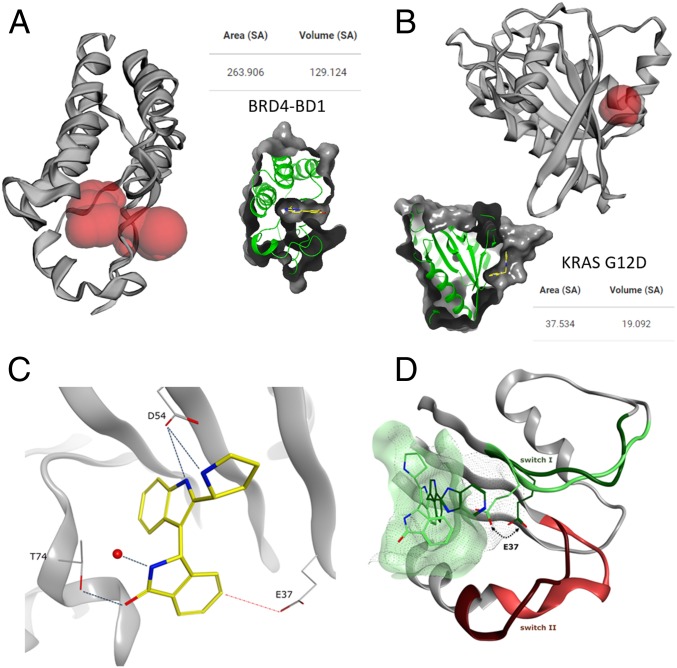
Analysis of protein pockets and X-ray structure of **15** in GCP-KRAS^G12D^. A Computer Atlas of Surface Topography of proteins (48) analysis of pockets in proteins for calculating the solvent-accessible surface area (Area SA) and volume (Volume SA) with a radius probe of 1.4 Å showing differences in pocket size for (*A*) BRD4-BD1 (PDB ID code 5M39) as an example of a highly druggable pocket and (*B*) KRAS G12D (PDB ID code 6GJ5) with a very small volume. Calculated pockets are shown in red. *Insets* show ligand binding in the respective pocket, for comparison reasons. (*C*) Polar interactions formed by **15** to T74, D54, and a conserved water. The ideal position of E37 for introducing a further polar interaction is highlighted with the red dotted line (PDB ID code 6GJ5). (*D*) Comparison of SI/II-pocket in GDP (PDB ID code 4EPV) and GTP-KRAS showing the significantly reduced pocket size in GTP-KRAS. The switch I and switch II regions are colored dark green and dark red in GDP-KRAS and light green and light red for GTP-KRAS, respectively.

The SI/II-pocket on KRAS (Fig. 2*B*) is around 7 times smaller in volume compared with the druggable acetyl lysine-binding pocket on the bromodomain containing protein BRD4 (Fig. 2*A*). As such, optimizing ligands for this small, shallow, and polar pocket represents a daunting challenge for medicinal chemistry. Our strategy to overcome this was to use structure-based drug design to precisely target polar interactions within the binding site while minimizing the ligand desolvation penalty (31). The design of molecules capable of forming an H bond to the side-chain hydroxyl of T74 was prioritized to gain further binding affinity, and the dihydroisoindolinone moiety linked with the S configuration to the 3 position of the indol-2-ylmethylamino fragments was chosen. Importantly, this compound series led to an affinity improvement of 5- to 10-fold (*SI Appendix*, Table S2), with the pyrrolidine analog **15** displaying an NMR K_D_ of 440 µM.

Given the significant improvement in binding potency of these compounds, we reverted to the use of the naturally occurring T35 construct, with NMR K_D_s being easily measured despite the reduction in visible cross-peaks for the G12D, C118S double mutant construct (*SI Appendix*, Table S2). C118S was introduced for stability reasons in NMR as described before (14). More importantly, we were successful in obtaining an X-ray structure of **15** in the new GCP-KRAS^G12D,C118S^ construct. As designed, the carbonyl oxygen of the dihydroisoindolinone forms a H bond to T74 at a distance of 2.4 Å, and H bonds involving the pyrrolidine and indole nitrogens of **15** are formed with D54 (Fig. 2*C*). In addition, a conserved water molecule is coordinated to the indolinone nitrogen. Interestingly, E37 blocks part of the SI/II-pocket in GCP-KRAS, compared with the GDP-KRAS structure 4EPV, even further reducing the size of the SI/II-pocket in the GTP form of KRAS (Fig. 2*D*). Important for further optimization was the observation that the side-chain position of E37 was ideally placed to form a fourth polar interaction with isoindolinones substituted at the 5 position.

### Discovery of the Micromolar RAS Inhibitor 18.

**Fig. 3.**
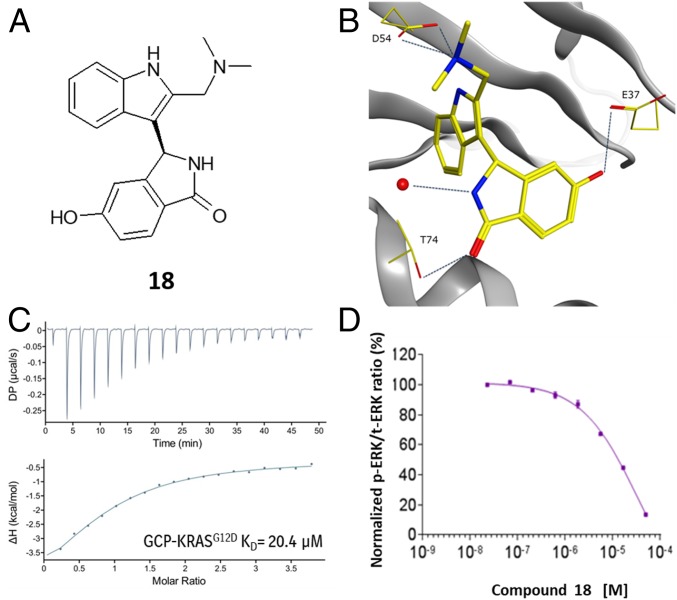
X-ray, biophysical and cellular data for **18**. (*A*) Chemical structure of **18**. (*B*) X-ray structure of **18** in GCP-KRAS^G12D^, highlighting the polar interactions formed with D54, T74, and E37 (PDB ID code 6GJ6). (*C*) ITC dose titration curve for **18** and GCP-KRAS^G12D^. (*D*) Meso Scale Discovery analysis of pERK levels in NCI-H358 cells after 2-h treatment of **18**.

Based on the X-ray structure of **15** bound to GCP-KRAS, we chose to introduce a phenolic oxygen as a H-bond donor at the 5 position of the isoindolinone to interact with E37. The 5-hydroxy-isoindolinone **18** (Fig. 3*A*) was 6-fold more potent than the nonphenolic matched molecular pair **17** (*SI Appendix*, Table S2), with an isothermal titration calorimetry (ITC) (32) K_D_ of 20 µM (Fig. 3*C*). X-ray crystallography confirmed the desired phenolic H bond to E37 at a distance of 2.7 Å and, all 3 previously discovered polar interactions to D54 and T74 were maintained (Fig. 3*B*).

Given the improved potency of **18** and the high conservation of the SI/II-pocket across RAS isoforms, selectivity was evaluated using ITC, and **18** was found to bind with similar affinity to both the GCP and GDP forms of KRAS, HRAS, and NRAS, including both mutant and wild-type KRAS (*SI Appendix*, Table S3 and Fig. S3). Compound **18** also showed biochemical inhibition of GTP-KRAS with SOS1 (EC_50_ = 33 µM; see *SI Appendix*, Table S4) using a time-resolved fluorescence energy transfer assay.

The cellular activity of **18** was evaluated in the lung cancer cell line NCI-H358, which bears a heterozygous KRAS^G12C^ mutation. Active RAS signals through RAF and MEK to induce phosphorylation of ERK (phospho-ERK). Phospho-ERK levels were quantified using a plate-based electrochemiluminescent assay (MESO SCALE DISCOVERY). A dose-dependent decrease in phospho-ERK levels was observed 2 h after treatment of NCI-H358 cells with **18**, leading to almost complete inhibition at 50 µM (Fig. 3*D*).

### Discovery of the Nanomolar RAS Inhibitor BI-2852 (1).

**Fig. 4.**
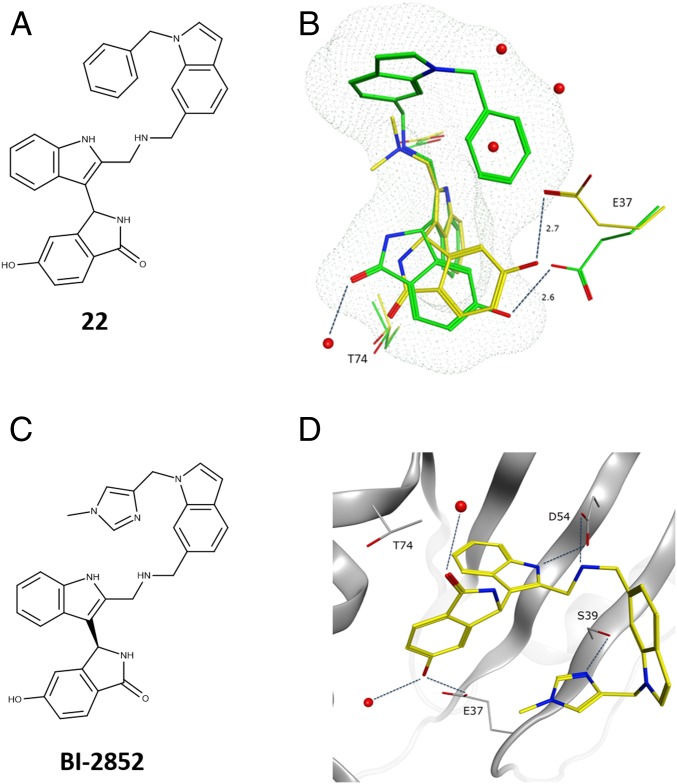
GCP-KRAS^G12D^ X-ray structures of compound **22** and **BI-2852** (**1**). (*A*) Chemical structure of **22**. (*B*) Overlay of the binding modes of **18** and **22**. The relative orientation of **18** and E37 in the X-ray with KRAS^G12D^ is depicted in yellow. The binding mode of **22**, E37, and T74 are depicted in green. Dotted mesh depicts the van der Waals radii around **22**, showing overlap with 3 waters from the X-ray structure of **18** (PDB ID code 6GJ8). (*C*) Chemical structure of **1**. (*D*) X-ray structure of **1** in GCP-KRAS^G12D^, highlighting the polar interactions formed with D54, T74, S39, and E37 (PDB ID code 6GJ7). The racemate **23** was used for soaking, and eutomer **1** was crystallized.

To further improve the binding affinity of the compounds, we explored a variety of substituents at the 2-methylamino position (*SI Appendix*, Table S4). While a benzyl substituent **19** showed no improvement in potency, indole substituted compounds **20** and **21** showed a 5-fold improvement in biochemical activity. Thus, we explored larger substituents at the pendant indole nitrogen and discovered that the racemic N-benzylindole derivative **22** (Fig. 4*A*, *SI Appendix*, Table S4) exhibited submicromolar activity in the GTP-KRAS::SOS1 fluorescence resonance energy transfer assay with an IC_50_ of 870 nM.

The X-ray structure of **22** in complex with GCP-KRAS showed that the N-benzylindole moiety folds back on itself to displace 3 water molecules present in this area of the pocket when unoccupied (Fig. 4*B*). Despite the side-chain rotation of E37 to avoid a clash with the benzyl group, the H bond with E37 was maintained. However, the direct interaction with T74 was lost, and the carbonyl forms a H bond to a water molecule instead. Due to the high lipophilicity (ClogP of 4.8), **22** displayed poor solubility (<1 µg/mL at pH 6.8) and, as such, did not constitute a molecule of sufficient quality for reliably investigating KRAS biology.

To overcome the solubility limitations of **22**, the chiral *N*-methyl imidazole derivative **1** (Fig. 4*C*) was prepared. This compound exhibited a significantly reduced ClogP of 2.6 and had a solubility of 18 µg/mL at pH 6.8 while maintaining nanomolar binding affinity to GTP-KRAS^G12D^ (K_D_ = 750 nM) as measured by ITC and with an IC_50_ of 450 nM in the Alpha Screen (*SI Appendix*, Tables S5 and S6). All interactions observed for **22** were maintained, with an additional H bond formed between the imidazole nitrogen and the side-chain oxygen of S39 at a distance of 2.7 Å (Fig. 4*D*). As observed for compound **18**, **1** also binds with similar affinity to KRAS, NRAS, and HRAS (*SI Appendix*, Fig. S4 and Table S5), with the exception of a small selectivity window (5- to 10-fold) to active KRAS^wt^ and inactive NRAS^wt^.

### Characteristics of the RAS Inhibitor BI-2852 (1).

**Fig. 5.**
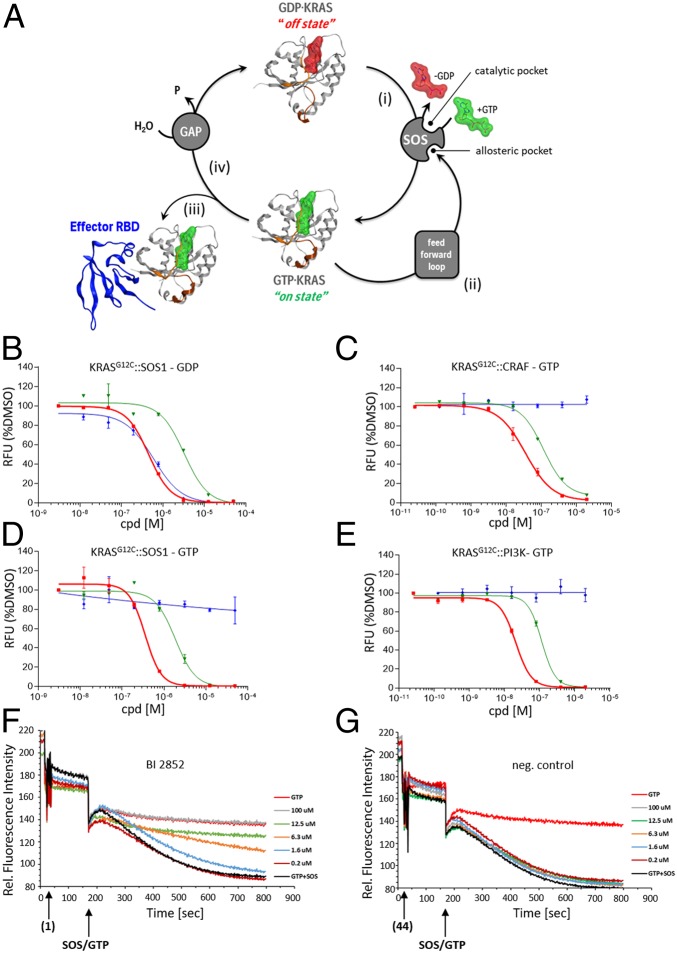
Biochemical assay dose–response curves for **BI-2852** (**1**), distomer **44**, and **ARS-1620**. (*A*) KRAS cycle is depicted with KRAS in Channing Der‘s “beating heart of cancer” orientation switching between its “off state” with the nucleotide GDP bound (red surface) and its “on state” with GCP bound (green surface). The 4 PPI intervention points in the KRAS cycle are shown. (*i*) The interaction between GDP-KRAS and the catalytic site of its GEF; here SOS is depicted. (*ii*) GTP-KRAS binding to the allosteric site of SOS which constitutes the feed forward loop. (*iii*) GTP-KRAS binding to downstream effectors; CRAF (in blue) is shown as an example. (*iv*) GTP-KRAS binding to GAPs. KRAS is depicted in gray, with the switch I region colored in orange and the switch II region in brown. (*B*−*E*) Biochemical assay dose–response curves for **1** (red), distomer **44** (green), and ARS-1620 (blue) for (*B*) GDP-KRAS^G12C^::SOS1 alpha screen assay, (*C*) GTP-KRAS^G12C^::CRAF alpha screen assay, (*D*) GTP-KRAS^G12C^::SOS1 alpha assay, and (*E*) GTP-KRAS^G12C^::PI3Kα alpha screen assay. All values shown are normalized to DMSO (= 100%) for better comparability. Error bars indicated show the SD of duplicates measured. Shown are representative examples of multiple repetitions with identical results. (*F* and *G*) The ability of test compounds to affect SOScat-catalyzed nucleotide exchange on RAS was assessed at several concentrations. The addition of SOScat and excess GTP (at 120 s) initiates the exchange of labeled boron-dipyrromethene-GDP (BODIPY-GDP) already loaded into RAS. The BODIPY-GDP to GTP exchange mediated by SOScat (black curve) is observed as a decrease in relative fluorescence units (RFUs) over time. While the negative control distomer **44** (*G*) shows no effect, increasing concentrations of **BI-2852 (**1**)** (*F*) show a slower decrease in RFU over time, representing a slower exchange rate. The highest concentrations show full inhibition of SOScat-mediated exchange, matching the rate in the absence of SOScat (red curve).

Using biochemical assays, we investigated whether **1** inhibited 3 of the 4 PPIs important for KRAS cycling (Fig. 5 *A*, *i*–*iii*). Namely, 1) GDP-KRAS interaction with the catalytic site of SOS (33), 2) GTP-KRAS interaction with the allosteric site of SOS (34), and 3) GTP-KRAS interaction with downstream effectors (CRAF and PI3Kα). We were unable to establish a biochemical assay for the fourth intervention point, namely, 4) GTP-KRAS interaction with its GAPs (Fig. 5 *A*, *iv*). As a reference compound for these assays, we used the KRAS^G12C^-specific covalent inhibitor **ARS-1620** (35). Compound **1** inhibited all 3 RAS cycle intervention points (1 to 3) in a dose-dependent manner (Fig. 5 *B*–*E*) in the range of 100 to 770 nM (*SI Appendix*, Table S6). **ARS-1620** displayed activity on the GDP-dependent KRAS^G12C^::SOS1 interaction, but was inactive in all GTP-dependent KRAS assays, in line with the lack of accessibility of the pocket when GTP is bound to KRAS. Moreover, when we exchanged the G12C mutation in KRAS against G12D, **ARS-1620** lost the ability to inhibit the GDP-dependent interaction, while **1** maintained the inhibitory function (*SI Appendix*, Table S6).

**Fig. 6.**
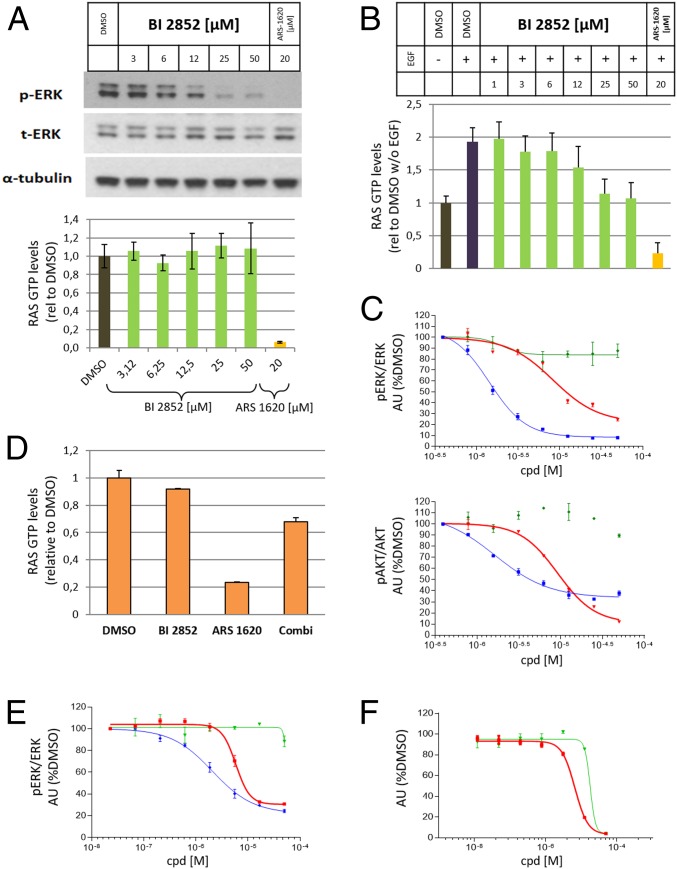
Cellular data for **BI-2852** (**1**), distomer **44**, and **ARS-1620**. (*A*) Western blot of pERK levels versus total ERK and alpha-tubulin under high serum conditions in NCI-H358 cells upon increasing doses of **1** and a fixed concentration of **ARS-1620** (*Upper*) and G-LISA assay measuring GTP-RAS levels under high serum conditions in NCI-H358 cells upon increasing doses of **1** and a fixed concentration of **ARS-1620** (*Lower*). (*B*) GTP-RAS levels measured with a G-LISA assay in NCI-H358 cells starved for 24 h in low serum conditions (−EGF), followed by 2-h treatment with increasing concentrations of **1** or 20 µM treatment of **ARS-1620** and then EGF addition. For quantification, RAS GTP levels without EGF and in the presence of DMSO were set to 1 (*A* and *B*). (*C*) pERK and pAKT levels in NCI-H358 cells starved for 24 h in low serum conditions (−EGF), followed by 2-h treatment with increasing concentrations of **1** (red), **44** (green), and **ARS-1620** (blue); then EGF addition were quantified. DMSO-treated samples after EGF stimulation were set to 100%. (*D*) GTP-RAS levels measured with a G-LISA assay in NCI-H358 cells with treatment of 50 µM compound **1**, 20 µM **ARS-1620** and simultaneous treatment of **1** and **ARS-1620**. For quantification, RAS GTP levels in the presence of DMSO were set to 1. (*E*) pERK dose–response curves for **1** (red), **44** (green), and **ARS-1620** (blue) in NCI-H358 cells under high serum conditions. (*F*) Antiproliferative dose–response curves for NCI-H358 cells in soft agar and low serum conditions for **1** (red) and **44** (blue). DMSO-treated samples were set to 100% (*E* and *F*). Error bars indicate SDs. Indicated experiments were performed 2 or more times with similar results.

Compound **1** also inhibits the rate of nucleotide exchange in a dose-dependent manner as measured by an SOS1 nucleotide exchange assay (36) (Fig. 5*F*). Despite this fact and a dose-dependent reduction of pERK, **1** does not change steady-state RAS GTP levels in NCI-H358 cells under high growth factor conditions, while the covalent GDP-KRAS^G12C^ inhibitor **ARS-1620** strongly reduced RAS-GTP levels (Fig. 6*A*). To demonstrate in cells that **1** inhibits SOS-catalyzed nucleotide exchange, we starved NCI-H358 cells for 24 h in low serum, causing a decrease of GTP-loaded KRAS. When we added **1** for 2 h and then added epidermal growth factor (EGF) to artificially increase RAS GTP levels, we observed a dose-dependent inhibition of the formation of GTP-loaded KRAS, which, at high concentrations, stayed at the levels of the dimethyl sulfoxide (DMSO)-treated sample (Fig. 6*B*). This result is in line with a blockade of the SOS-catalyzed conversion of KRAS from GDP to GTP. **ARS-1620** reduced the RAS GTP levels even below the DMSO control. Analysis of downstream signaling events revealed that, under the described conditions, pERK and pAKT levels were dose-dependently reduced (Fig. 6*C*). Next, we explored whether **1** could also interfere with the GAP-catalyzed conversion of GTP-loaded KRAS to inactive GDP-bound KRAS in cells, as we were unable to investigate this biochemically. To our knowledge, there is no physiological condition available to rapidly convert GTP-KRAS to the inactive form; hence we made use of **ARS-1620**, which, when applied in sufficient concentration, causes a near-complete depletion of active GTP KRAS^G12C^. When we incubated NCI-H358 cells with 50 µM **1** for 1 h, as previously observed, no change in GTP-KRAS was observed. In contrast, 20 µM **ARS-1620** caused an 80% reduction of the GTP-bound RAS pool. When the 2 compounds were combined, only a 30% reduction of GTP-RAS was observed, which is in line with inhibiting the binding of GAPs with KRAS (Fig. 6*D*).

The cellular signaling activity of **1** was tested in NCI-H358 cells. NCI-H358 cells cultured under high growth factor conditions were treated with increasing concentrations of **1** and **ARS-1620** for 2 h. A dose-dependent inhibition of pERK relative to total ERK (EC_50_ = 5.8 µM) was observed after treatment with **1**, with a similar, albeit more extended, inhibition of pERK being observed for **ARS-1620** (Fig. 6*E*). A rebound in pERK inhibition was observed (*SI Appendix*, Fig. S5) similar to what was observed for BRAF inhibitors in BRAF^V600E^ mutated cell lines (37). In contrast to low serum conditions, no reduction of pAKT levels was observed under high serum conditions in NCI-H358 cells. We then assessed whether the observed pERK reduction after 2 h would lead to an antiproliferative effect on cells. We plated NCI-H358 cells in soft agar and low serum conditions and indeed observed a dose-dependent antiproliferative effect of **1** at an EC_50_ of 6.7 µM (Fig. 6*F*). No antiproliferative effect was observed under standard 2D culture conditions, in line with recent observations that KRAS proliferation inhibition is predominantly measurable under 3D, nonadherent conditions (35).

To convince ourselves that the effects of **1** were due to direct KRAS inhibition and not unspecific effects, the properties of the distomer **44** were investigated. The distomer **44** inhibited the PPIs between the GTP and GDP forms of KRAS ∼10-fold more weakly than the eutomer **1** (Fig. 5 *B*–*E* and *SI Appendix*, Table S6). The distomer **44** also did not inhibit SOS1-catalyzed nucleotide exchange (Fig. 5*G*). This difference in activity is consistently maintained in the cellular assays with no pERK reduction observed at concentrations up to 50 µM (Fig. 6*E*) and a significant antiproliferative effect observed only at 50 µM (Fig. 6*F*). Further, we have tested **1** and **44** in 4 BRAF(v600E) cell lines which signal in a RAS-independent manner (38). No antiproliferative effect was observed for **1** and **44** in any of the 4 cell lines under low serum, soft agar, or high serum, adherent conditions, and no inhibition of pERK was observed (*SI Appendix*, Fig. S6). This clearly demonstrates that **BI-2852** does not exhibit off-target antiproliferative effects. Taken together, these data support the interpretation that the biochemical and cellular effects observed for **1** are the result of direct inhibition of KRAS.

## Discussion

Here, we describe the discovery of **1**, an inhibitor of both the active and inactive form of KRAS, with nanomolar binding affinity to the SI/II-pocket, a small, shallow, and polar pocket deemed by many to be “undruggable.” Compound **1** is the first RAS inhibitor for the SI/II pocket with KRAS-driven cellular activity, displaying low micromolar pERK modulation and antiproliferative effects on a KRAS mutant cell line. Recent compounds (16, 17) claiming cellular activity do not provide negative control data and display antiproliferative effects under 2D cell culture conditions which should not be interpreted as KRAS-driven, given that KRAS antiproliferative effects are predominantly only observed under 3D, nonadherent conditions (35).

Fragment screens delivered hits in the millimolar binding affinity range which were optimized using structure-based design. Although highly resource-intensive, the application of NMR to measure dissociation constants for newly designed compounds in the millimolar range served as an important method for establishing structure activity relationships. Guided by the X-ray structures of cocomplexes, we were able to optimize binding, as evidenced by the discovery of the isoindolinone **18**, which bound to GCP-KRAS^G12D^ at 20 µM by ITC and also displayed inhibition of the key PPI with KRAS in a similar range. This allowed us to switch from NMR K_D_ measurements to biochemical PPI assays to further optimize the potency of the compounds.

Obtaining 3D crystallographic information on RAS proteins in the active form has been historically limited. The development of a reliable procedure to produce >10 mg/L of purified GCP-KRAS was instrumental in enabling crystallography, which, in turn, revealed critical information on the binding of the ligands to the SI/II-pocket of GCP-KRAS. Compound **1** maintained the polar interactions to D54 and E37 also addressed by **18** and, in addition, formed a H bond to S39 and displaced 3 water molecules, which are presumably responsible for the >100-fold improvement in potency. It should be noted that T74, which improves potency by 5- to 10-fold (*SI Appendix*, Tables S1 and S2), is not yet addressed by **1** and that **1** still contains 7 rotatable bonds. This highlights the potential for significant improvement beyond the current potency of **1** (e.g., IC_50_ of 180 nM for the PPI between active KRAS^G12D^ and CRAF) and indicates that the SI/II-pocket is indeed druggable.

Triple *RAS* knockout mice are not embryonically viable but can be rescued by reintroduction of an HRAS transgene, indicating functional redundancy among the RAS family (39) and suggesting that sparring at least one wild-type RAS isoform will be needed for a RAS drug. As the SI/II-pocket is conserved on both the inactive and active forms of all RAS isoforms, obtaining sufficient selectivity presents an additional significant challenge to drugging this pocket. Interestingly, **1** demonstrates a 10-fold selectivity of binding to GCP-KRAS^wt^ (*SI Appendix*, Table S5) which translates to a 4-fold selectivity of inhibition biochemically (inhibition of KRAS^wt^ versus KRAS^G12C^ binding to CRAF) (*SI Appendix*, Table S6). The relative lack of selectivity versus the KRAS^G12D^::CRAF is expected due to the 5-fold weaker affinity of KRAS^G12D^ for CRAF, while KRAS^G12C^ and KRAS^wt^ maintain the same affinity for the RAS binding domain of CRAF (40). Also, a weaker affinity to GDP-NRAS was observed for **1**. Together, this suggests that, despite the high conservation of the SI/II-pocket, it might be possible to design molecules with sufficient RAS isoform selectivity.

The SI/II-pocket is involved in interactions with GEFs (41), GAPs (42), and downstream effectors (43, 44), and we provide evidence that compound **1** inhibits all of these PPIs. Functionally, **1** inhibits SOS1-catalyzed exchange of GDP-KRAS to GTP-KRAS as well as GAP-catalyzed exchange of GTP-KRAS to GDP-KRAS, which results in no net change in cellular GTP-RAS levels upon treatment. **E37** on switch II, to which the phenolic oxygen of **1** H-bonds, is also an important residue for RAS binding to downstream effectors (45), GEFs (41, 46), and GAPs (47), explaining mechanistically how **1** inhibits the binding of multiple key RAS interactions partners.

Compound **1** reduced pERK and pAKT levels in a dose-dependent manner in NCI-H358 cells, leading to an antiproliferative effect in NCI-H358 cells under nonadherent, low serum conditions. The effects of **1** were confirmed to be KRAS-driven and not off-target through the consistent data generated for the 10-fold less active distomer **44** and through the absence of any effects on BRAF(V600E) cell lines. We expect **BI-2852** to serve as a useful chemical probe for the study of RAS biology in an in vitro setting, and it is available to the scientific community (https://opnme.com/molecules/kras-bi-2852). **BI-2852** is also an ideal starting point for the design of more-potent and selective RAS inhibitors.

## Supplementary Material

Supplementary File
