# Supplementary material for: Drugging an undruggable pocket on KRAS
Source: Proc Natl Acad Sci U S A. 2019 Jul 22;116(32):15823–9. doi: 10.1073/pnas.1904529116 (PMC6689897; doi:10.1073/pnas.1904529116)
Supplement: Supplementary File [file pnas.1904529116.sapp.pdf]

PNAS
www.pnas.org

Supplementary Information for

Drugging an undruggable pocket on KRAS

Dirk Kessler[a,1], Michael Gmachl[a,1], Andreas Mantoulidis [a,1], Laetitia J Martin [a,1], Andreas Zoephel[a], Moriz Mayer[a], Andreas Gollner[a], David Covini[a], Silke Fischer[a], Thomas Gerstberger[a], Teresa Gmaschitz[a], Craig Goodwin[c], Peter Greb[a], Daniela Häring[a], Wolfgang Hela[a], Johann Hoffmann[a], Lyne Lamarre[a], Jale Karolyi-Oezguer[a], Petr Knesl[a], Stefan Kornigg[a], Manfred Koegl[a], Roland Kousek[a], Franziska Moser[b], Silvia Munico-Martinez[a], Christoph Peinsipp[a], Jason Phan[c], Jörg Rinnenthal[a], Jiqing Sai[c],Christian Salamon[a], Yvonne Scherbantin[a], Katharina Schipany[a], Renate Schnitzer[a], Andreas Schrenk[a], Bernadette Sharps[a], Gabriella Siszler[a], Qi Sun[c], Alex Waterson[c], Bernhard Wolkerstorfer[a], Markus Zeeb[b], Mark Pearson[a], Stephen W. Fesik[c] and Darryl B. McConnell[a,2]

Darryl B. McConnell
Email: darryl.mcconnell@boehringer-ingelheim.com

This PDF file includes:

Supplementary text
Figures S1 to S6
Tables S1 to S7
References for SI reference citations
Author contributions

www.pnas.org/cgi/doi/10.1073/pnas.1904529116

Supplementary Text

Microscale Thermophoresis (MST)

MST experiments were performed with a modified and fully automated Monolith NT.015 (NanoTemper Technologies, Munich, Germany). In collaboration with Nanotemper Technologies the in-house implementation combined the MST device with a Hamilton Microlab Star pipetting system (Hamilton Robotics, Bonaduz, Switzerland) equipped with an in-house developed tilting station. Each capillary was filled by dipping into each well of the vertically tilted 384-well plate (Greiner PP, small volume, deep well from Greiner Bio-One, Frickenhausen, Germany) using a pneumatic gripper (Schunk, Lauffen/Neckar, Germany), which was attached to a pipetting channel. Capillaries were individually transferred to a capillary holder. The latter with the set of 16 capillaries was then loaded on the capillary tray of the instrument and subsequent data acquisition.

Fluorescence labeling of GCP-KRAS^{G12D} with the NT647 dye was carried out according to the manufacturer's protocol of the Monolith NT.115 Protein Labeling Kit RED-NHS (NanoTemper Technologies, Munich, Germany). Assay development comprised the optimization of protein concentration, buffer conditions, MST capillaries as well as the strength of the temperature gradient (IR laser power) using an in-house positive control to achieve a reliably detectable change in the thermophoretic mobility (ΔF_{norm}). Fragments were diluted in 30 µL assay buffer (20 mM HEPES pH 7.4, 150 mM NaCl, 2 mM $MgCl_2$, 1 mM TCEP, 0.05% Tween-20) with a Hamilton Microlab Star liquid handling system and 10 µL of labeled GCP-KRAS^{G12D} was added just-in-time prior to data acquisition to achieve equal incubation times. Final concentrations were 100 nM fluorescently labeled GCP-KRAS^{G12D}, 500 µM fragment and 2% DMSO. Standard treated capillaries, IR laser powers of 20% and 70% (30s laser on time) and a LED intensity of 50% were used.(1) Capillary positions 1 and 16 were used as DMSO negative controls, whereas capillaries 2-14 contained seven fragments in duplicates. Data was analyzed with the NanoTemper Analysis software version 1.2.205 from which ΔF_{norm} values ($\Delta F_{norm}=F_{hot}/F_{cold}$) as well as the initial fluorescence was exported. All MST traces were inspected manually and irregular traces (e.g. fluorescence quenching, protein aggregation) were discarded. Mean values of duplicates were calculated and compared to either the mean value of the DMSO negative control in the individual acquisition cycle or the mean value of the DMSO control of the respective 384-well screening plate. Integrity of labeled GCP-KRAS^{G12D} throughout screening was monitored by the same positive control as used for assay development. Fragments were classified as hits if $\Delta\Delta F_{norm} \geq \Delta F_{norm}(2sd\ DMSO)$ with $\Delta\Delta F_{norm} = |\Delta F_{norm}(compound)-\Delta F_{norm}(DMSO)|$.

NMR spectroscopy

STD-NMR experiments were carried out on a Bruker Avance II 600 MHz instrument equipped with a QCI cryogenic probe and z-gradients. Samples were freshly prepared just-in-time and NMR sample tubes were filled with an in-house customized Tecan Freedom Evo liquid handler. Transfer of the samples (140 µL in 2.5 mm NMR tubes) to the NMR magnet was achieved with the Bruker Sample Rail system. For the STD experiment, a Gaussian pulse train was used for selective irradiation (duration of 3 s) at -0.2 ppm (on-resonance spectrum) or at 60 ppm (off-resonance spectrum), respectively. Protein signals were suppressed by a 30 ms spin-lock pulse. Both spectra were recorded in an interleaved mode and subtracted after acquisition to yield the

difference spectrum. Mixtures of four fragments (each at 250 μM) were incubated in 25 mM Tris-d11, 100 mM NaCl, 2 mM $MgCl_2$, 0.5 mM TCEP pH 7.4 in D_2O at 298K with a mixture of GCP-$KRAS^{G12D}$ and GDP-$KRAS^{G12V}$ (each 4 μM). Binders were identified by comparing pre-recorded reference spectra with the STD difference spectrum.

Deconvolution and confirmation of primary FBS hits obtained from STD-NMR and MST was performed using 2D $^1H/^{15}N$ TROSY NMR experiments(2) collected on a Bruker Avance III 600 MHz spectrometer equipped with a 5 mm z-gradient TCI cryo-probe. Samples were prepared directly before data acquisition using a Tecan Freedom Evo pipetting system and delivered by a Bruker Sample Rail to the magnet. Each sample contained either 50 μM ^{15}N labeled GDP-$KRAS^{G12D}$ or GCP-$KRAS^{G12D}$ in 25 mM Tris-d11, 100 mM NaCl, 2 mM $MgCl_2$, 0.5 mM TCEP pH 7.4 and 8% (v/v) D_2O. Proteins were incubated with 500 μM fragment in a 2.5 mm NMR tube at 298K and 1% d6DMSO. Spectra were recorded with 32 transients and 64 data points in the indirect dimension. Processing and analysis were accomplished with Topspin 3.0 (Bruker BioSpin). Binders were identified by manual comparison of spectra in the presence of a fragment and the respective reference spectrum. All confirmed binders to ^{15}N labeled GDP-$KRAS^{G12D}$ show also chemical shift perturbations in the presence of ^{15}N labeled GDP-$KRAS^{G12V}$ and are therefore non-selective.

Measurement of dissociation constants K_D for the compounds synthesized. NMR was initially the only method that could provide K_Ds for the weakly binding molecules to support compound optimization. $^1H/^{15}N$ SoFast HMQC experiments(3) were recorded in 3mm NMR tubes (170μL filling) at a protein concentration of 70-100μM. Spectra were recorded on a Avance III 700MHz spectrometer equipped with a cryogenically cooled 5mm TCI probe and 48 scans, 128 f1 increments and 2k data points in f2. Total acquisition time was 22 minutes. 4 – 6 titration points were performed with individual samples for each titration point made from 50mM DMSO-d^6 stock solutions. Total DMSO-d^6 concentration was kept constant by backfilling every sample to a total of 4% DMSO. K_Ds were calculated from the differences in chemical shift induced by the small molecules upon interacting with the protein. Average chemical shifts from the ^1H and ^{15}N dimension were calculated according to the following equation:

$$\Delta\delta_{obs} = \sqrt{[\delta_H^2 + (\alpha \cdot \delta_N)^2]} \text{ with } \alpha \text{ set to } 0.14$$

The $\Delta\delta_{obs}$ values at the respective ligand concentrations and the total concentration of protein allow a fit to the K_D value of the ligand as long as it is binding under fast exchange conditions as was the case for most of the compounds tested.(4) Usually $\Delta\delta_{obs}$ values of 3 to 5 different resonances were used and an average K_D determined. The following equation was used to determine the individual K_Ds:

$$\Delta\delta_{obs} = \Delta\delta_{max}\left[([P]_t + [L]_t + KD) - [([P]_t + [L]_t + KD)^2 - 4([P]_t[L]_t)]^{1/2}\right]/2[P]_t$$

ITC assays

Calorimetric experiments were performed on a Microcal PEAQ-ITC calorimeter (MicroCalTM, LLC Northampton, MA). Protein solutions were measured in 20 mM HEPES, 150 mM NaCl, 3% DMSO, pH 7.5. All measurements were carried out at 25°C. Titrations were performed in inverse mode. The cell was loaded with compound solutions in the range of 10 to 100 μM. All injections were performed using an initial injection of 0.5 μL followed by 19 injections of 2 μL of protein in the range of 130-500 μM. The data were analyzed with the MicroCal PEAQ-ITC analysis software package. The first data

point was excluded from the analysis. Thermodynamic parameters were calculated ($\Delta G = \Delta H - T\Delta S = -RTlnK_B$, where ΔG, ΔH and ΔS are the changes in free energy, enthalpy and entropy of binding, respectively).

Protein purification, nucleotide exchange and crystallization

The KRAS construct, based on uniprot identifier P01116-2 (KRAS4-B) amino acids 1-169, was originally obtained from the Structural Genomics Consortium (SGC, Oxford) in a pET28 vector. KRAS constructs (G12D; G12D, C118S; G12D, C118S, T35S; and G12V) were expressed with an N-terminal His6-tag followed by a TEV (tobacco etch virus protease) cleavage site. Mutations were inserted by QuikChange II Site-Directed Mutagenesis Kit (Agilent) and confirmed by sequencing. The Q61H mutation, originally present in the construct, was reverted to Q. For crystallization, tags were removed by cleavage with TEV leaving an additional glycine at the N-terminus of the proteins after cleavage with TEV.

NRAS wt: The expressed protein corresponds to amino acids 1-172 of reference sequence P01111 (uniprot) with an additional glycine at the N-terminus after cleavage of the His-tag. A surface cysteine (C118) was mutated to serine for stability reasons.

HRAS wt: The expressed protein corresponds to amino acids 1-166 of reference sequence P01112 (uniprot) with an additional glycine at the N-terminus after cleavage of the His-tag.

Expression and Purification

KRAS gene constructs were expressed in E. coli BL21 (DE3) cells in Terrific Broth (TB) media, induced with 0.2 mM IPTG at 18°C for 18 h.

Cells were harvested by centrifugation and stored at -80 °C. Cell pellets were extracted by sonication in 20 mM Tris, 500mM NaCl, 5 mM imidazole, 0.5% 3-[(3-cholamidopropyl)dimethylammonio]-1-propanesulfonate (CHAPS), containing protease inhibitors (cOmplete, EDTA-free, Merck), pH 8.0 (extraction buffer). Cell debris was removed by centrifugation for 45 min at 16,000 rpm in a JA 16,250 rotor (Beckman) at 4°C. Recombinant KRAS was loaded onto Ni-NTA resin (Qiagen) by incubation for 2.5 h on ice. The slurry was loaded into a column and washed extensively with extraction buffer (without protease inhibitors). KRAS was eluted with 0.25 M imidazole in extraction buffer, desalted into 20 mM HEPES, 150 mM NaCl, pH 7.5 (HiPrep Desalting, GE Healthcare) and supplemented with 1 mg GDP per 20 mg protein and 5 mM $MgCl_2$. The His-tag was cleaved off by incubation with TEV protease overnight at 4°C. On the following day the cleaved His-tag was removed by passing the protein solution through a Ni-NTA column (HisTrap, GE Healthcare).

KRAS protein was then further purified by gel filtration chromatography (HiLoad 75S, GE Healthcare) in 20 mM HEPES, 150 mM NaCl, pH 7.5. The purity of the KRAS preparations was checked by SDS-PAGE electrophoresis and pure fractions were pooled. Protein solutions were then concentrated in a pressurized stir-cell to >1 mM and used either as the GDP-bound form or underwent nucleotide exchange (see below) for generation of GCP-bound forms.

Similar protocols were used for preparation of NRAS and HRAS forms.

Nucleotide Exchange

Nucleotides were exchanged by a modification of the protocol devised by Eberth et al.(5) In brief: 1 U/mg protein of agarose bead-coupled alkaline phosphatase and a 1.5

molar excess of GppCp was added to concentrated (1 mM) KRAS (GDP) after addition of 0.2 M $(NH_4)_2SO_4$, 1 mM $ZnCl_2$ (final concentrations) to the KRAS preparation with rapid mixing. The reaction mix was incubated for 3 h at 4°C. After 3 hours, another 1 U agarose bead-coupled alkaline phosphatase aliquot and GppCp was added, corresponding to a 1.5 molar excess over KRAS. After another 3 hours an additional aliquot of agarose bead-coupled alkaline phosphatase and GppCp was added. The mixture was then incubated for 18 h at 4°C. The solution was centrifuged at 1500 x g, 4°C to remove the alkaline phosphatase beads. The mixture was then passed through a HiPrep Desalting 26/10 (GE Healthcare) column equilibrated in 20 mM Tris, 2 mM $MgCl_2$, 2 mM DTT, pH 7.5 and the protein fraction collected. The degree of nucleotide exchange was analyzed by reversed-phase HPLC.

Protein Crystallization

Protein crystallization was done using the hanging drop method by mixing 2.0 µL of apo KRAS G12D (30 mg/mL in 20 mM Tris, 2 mM $MgCl_2$, 2 mM DTT, pH 7.5) and 3-5 fold excess of compound. Crystallization conditions for the respective co-crystallization trials were: 1) compound **15**: 30% PEG3350, 0.2 M potassium acetate; 2) **18**: 20% PEG3350, 0.2M ammonium nitrate; 3) **22**: Molecular Dimensions Morpheus Screen with 30% Precipitant Mix1, 0.1M Morpheus buffer system 2 pH 7.5 and 10% nitrate phosphate sulfate mix; 4**) BI 2852**: 25% PEG3350, 0.2M ammonium sulfate and 0.1M bis-TRIS buffer at pH 6. Crystals grew within a few days to a final size of 100-200 µm and were frozen in liquid nitrogen with 25% ethylene glycol as cryo protectant. Data were collected either at a homelab source (Rigaku compact homelab source with a Saturn944+ detector; wavelength 1.5418 Å) or at the SLS beam line X06SA (Swiss Light Source, Paul Scherrer Institute; wavelength of 1 Å using the PILATUS 6M detector).

Images were processed with autoPROC.(6)

The resolution limits were set using default autoPROC settings using STARANISO for anisotropic resolution cut-off for compounds **2-4**. The structures were solved by molecular replacement using the KRAS structure 4EPV as a search model. Subsequent model building and refinement was done using standard protocols using CCP4, (7) (Collaborative Computational Project, Number 4) COOT(8) and autoBUSTER.(9)

For **15** PDB Code 6GJ5 the unit cell parameters were a = 42.07 Å, b = 40.04 Å, c = 94.28 Å and α, γ = 90° and β = 101,1°, the resolution = 1.49 Å, data and the structure was refined to Rwork and Rfree values of 18.43 % and 20.29 %, respectively, with 98.1 % of the residues in Ramachandran favoured regions as validated with Molprobity.(10)

For **18** PDB Code 6GJ6 (unit cell: a = 85.9 Å, b = 85.9 Å, c = 47.525 Å, α, γ = 90° and β = 120°, resolution = 1.76 Å) was refined to R/Rfree = 18.4/21.1 % with 96.86 % of the residues in Ramachandran favoured regions.

For **22** PDB Code 6GJ7 (unit cell: a = 41.8 Å, b = 116.7 Å, c = 91.8 Å, α, β, γ = 90°, resolution = 1.67 Å) was refined to R/Rfree = 22.6/25.7 % with 96.34 % of the residues in Ramachandran favoured regions.

For **BI 2852** PDB Code 6GJ8 (unit cell: a = 41.6 Å, b = 116.84 Å, c = 91.48 Å, α, β, γ = 90°, resolution = 1.65 Å) was refined to R/Rfree = 17.6/19.8 % with 97,01 % of the residues in Ramachandran favoured regions.

Statistics for data collection and refinement can be found in Table S7.

Stereo images (wall-eye stereo and cross-exe stereo) can be found in Figure S2.

The coordinates and structure factors of the structures have been deposited at the Protein Data Bank with the accession codes PDB Code **6GJ5, 6GJ6, 6GJ7, 6GJ8**.

Alpha Screen Assays

Measurements of various protein–protein interactions were performed using the Alpha Screen technology developed by Perkin Elmer. Recombinant RAS proteins (H-, N-, K-RAS variants; all KRAS variants are based on KRAS isoform 4B (uniprot id P01116-2); KRAS (G12D) 1-169, N-terminal 6His-tag, C-terminal avi-tag was from Xtal BioStructures, Inc., KRAS (G12C) 1-169, C-terminal avi-tag, biotinylated, mutations: C51S, C80L, C118S, NRAS(wt) 1-172, C-terminal avi-tag, biotinylated; HRAS(wt) 1-166, C-terminal avi-tag, biotinylated). Biotinylation was performed *in vitro* with recombinant BirA biotin-protein ligase as recommended by the manufacturer (Avidity LLC, Aurora, Colorado, USA). Interacting proteins such as SOS1 (564-1049, N-terminal GST-tag, TEV cleavage site), cRaf (1-303, N-terminal GST-tag, TEV cleavage site) and PI3KA- RBD (160-317, N-term-HIS_GST-tag) were expressed as glutathione S transferase (GST) fusions. Accordingly, the Alpha Screen beads were glutathione coated Alpha Lisa acceptor beads (Perkin Elmer AL 109 R) and Alpha Screen Streptavidin conjugated donor beads (Perkin Elmer 6760002L). Nucleotides were purchased from Sigma (GTP #G8877, GDP #G7127), Tween-20 from Biorad (#161-0781). All interaction assays were carried out in PBS, containing 0.1% bovine serum albumin, 0,05% Tween-20 and 10 μM of the corresponding nucleotide. Assays were carried out in white ProxiPlate-384 Plus plates (Perkin Elmer #6008280) in a final volume of 20 μL. In brief, biotinylated RAS proteins (10 nM final concentration) and GST-SOS1, GST-PI3K or GST-CRAF (10 nM final) were mixed with glutathione acceptor beads (5 μg/mL final concentration) in buffer, containing the corresponding nucleotides (GDP or GTP for assays containing SOS1, only GTP for interaction assays containing PI3K or CRAF) and were incubated for 30 min at room temperature. After addition of streptavidin donor beads (5 μg/mL final concentration) under green light, the mixture was further incubated for 60 min in the dark at room temperature. Single oxygen induced fluorescence was measured at an Enspire multimode plate reader (Perkin Elmer) according to the manufacturer's recommendations. Data were analyzed using the GraphPad Prism data software.

Cell Culture

NCI-H358 cells (ATCC HTB-182, Lot# 60322588) were grown in cell culture flasks (175 cm^2) using RPMI medium supplemented with 10% fetal bovine serum. Cultures were incubated at 37 °C and 5% CO_2 in a humidified atmosphere, with medium change or subcultivation 2-3 times a week. NCI-H23 cells (ATCC® Number: CRL-5800™) were grown in cell culture flasks (175 cm^2) using RPMI medium supplemented with 10% fetal bovine serum. Cultures were incubated at 37 °C and 5% CO_2 in a humidified atmosphere, with medium change or subcultivation 2-3 times a week. In case starvation conditions were used in combination with EGF stimulation, cells were kept in medium containing 2% FBS for 24h before compound addition for 2 hours followed by EGF stimulation (50ng/mL) for 10 minutes.

Control cell lines (BRAF V600E mutated) were A375 (ATCC® CRL-1619™, grown in DMEM 10% FCS), RKO (ATCC® CRL-2577™, grown in EMEM 10% FCS), Colo 201

(ATCC® CCL-224™; grown in RPMI 10% FCS) and SK-MEL-28 (ATCC® HTB-72™, grown in EMEM 10% FCS). Cell cultures were incubated at 37 °C and 5% CO_2 in a humidified atmosphere, with medium change or subcultivation 2-3 times a week.

Immunoblotting

Cells were lysed in 1x lysis buffer (Cell Signaling #9803) and protein concentration normalized to 10µg/µL. Proteins were separated by SDS-Page and transferred to Nitrocellulose membranes (BioRad) according to standard protocols. Membranes were immunoblotted with antibodies against pERK$^{T202/Y204}$ (Cell Signaling #2101), total ERK (Milipore #06-182) and Tubulin (Cell Signaling #2144) in 5% BSA in TBST blocking buffer. After primary antibody incubation membranes were incubated with anti-rabbit IgG secondary antibody (Dako #P0448) and detected with ECL (GE Healthcare Life Science #RPN2106).

Compound treatment of cells

Synthesized or purchased (ARS 1620) compounds were dissolved in 100% DMSO and stored at a concentration of 10mM at -20°C. Compounds were either serially diluted in medium or directly added to reach the intended final concentration. DMSO was added to control reactions as reference to keep the final DMSO concentration in all samples equal.

2D proliferation assay:

Cells were plated in 96 well plates (1500 cells per well) in the corresponding medium containing 10% FCS. The next day, compounds (Stock: 10mM in 100% DMSO), serially diluted in medium were added starting at 50µM with 1:5 dilutions. Cells were incubated at 37°C and 5% CO_2 in a humidified atmosphere for 3 days. Quantification of living cells was performed using the cell titer glow reagent (Promega) according to the manufacturer's recommendation. Luminescence was read in a 2030 VICTOR X5 (Perkin Elmer). Data were fitted by iterative calculation using a sigmoidal curve analysis program based on GraphPAD Prism with variable hill slope.

Soft Agar proliferation assay in low serum:

The assay set-up was composed of a bottom layer consisting of 90 µL medium including 1.2% agarose, a cell-layer consisting of 60 µL medium including 0.3% agarose and a top-layer consisting of 30 µL medium including the test compounds (without agarose). 96 well plates were from Corning (96-well Ultra low binding plates #CLS2474-24EA). For preparation of the bottom layer, 4% agarose (microwave-heated, Gibco # 18300-012) was mixed with culture medium (incl. 2% FBS, HyClone # SH30071.03) to a final dilution of 1.2% agarose in medium. Each 96 well was filled with 90 µL of the bottom layer suspension and cooled to room temperature for about 1h. For the cell-layer cells were trypsinized, counted and plated in 60 µL culture medium containing 2% FCS including 0.3% agarose (1500 cells per well). After cooling to room temperature for ~1h, the plates were incubated over night at 37°C and 5% CO_2 in a humidified atmosphere.

The next day the compounds (30µL of serial dilutions) were added in triplicates. The concentration of the test compounds covered the range between 10 micro molar and 0.13 nano molar minimum. Compounds (Stock: 10mM in 100% DMSO) were diluted in medium. Cells were incubated at 37°C and 5% CO_2 in a humidified atmosphere for 14 days. For detection of viable cells Alamar Blue (#DAL1100CSTM1, Invitrogen) dye was added according to manufacturer's instructions. Fluorescence intensity was determined using a fluorescence reader (2030 VICTOR X5, Perkin Elmer). The excitation wavelength was 544/15 nm, emission 590 nm. Data were fitted by iterative calculation using a sigmoidal curve analysis program based on GraphPAD Prism with variable hill slope.

MAPK pathway signaling (pERK, pAKT quantification)

MSD 96-well multispot assays for Phospho/Total ERK1/2 and Phospho(Ser473)/Total AKT whole cell lysate kits (Meso Scale Discovery, Gaithersburg, MD, #K15107D, #K15100D) were used according to the manufacturer's instructions. Briefly, 50.000 cells were seeded per well and grown in 96 well plates. Cells were washed in ice cold PBS and lysed in 50µL ice-cold lysis buffer supplemented with the provided protease inhibitors. MSD plates were blocked for 1h, washed and 40µL of lysate was added in duplicates to wells and incubated at room temperature for 3 hours while shaking at 400rpm. To measure the amount of phosphorylated AKT or ERK, the specific primary antibody was added for 1 hour. After washing, read buffer was added and the plates were measured on a SECTOR 6000 instrument (Meso Scale Discovery). Samples were normalized to total ERK or total AKT values, respectively.

RAS GTP detection (Ras G-LISA Activation assay):

Ras-GTP levels were analyzed using a Ras G-LISA assay kit (Cytoskeleton Inc., Denver, CO, USA, #BK131) according to the manufacturer's instructions. Briefly, 600.000 cells were seeded in 6 wells and grown to 70% confluence. Cells were washed with ice-cold PBS and lysed in 80µL ice-cold lysis buffer supplemented with the provided protease inhibitor cocktail. Lysates were quickly frozen in liquid nitrogen and stored at -80°C until further usage. After normalizing protein concentration, 40µg of protein was added in duplicates to wells of the Ras G-LISA plate coated with Ras GTP-binding protein and incubated at 4°C for 30 minutes while shaking at 400rpm. After washing, antigen presenting buffer was added for 2 minutes. To measure bound Ras GTP levels, wells were subsequently incubated with an anti-Ras primary antibody (1:50) followed by a HRP-labeled secondary antibody (1:500) and finally by adding a HRP detection reagent. Absorbance was measured by 490nm using an EnSpire Multimode Reader (Perkin Elmer). Background was determined by a negative control well and subtracted from all samples.

KRASG12D::SOS1 GTP TR-FRET assay

This assay was used to identify compounds which competitively interact with the binding of KRAS G12D to SOS1 in the presence of GTP.

The following binding partners have been used in this assay. Biotinylated KRAS G12D protein corresponding to KRAS (amino acids 1-169, with the following changes to the natural protein: G12D) was expressed in E. coli with a carboxy-terminal Avi tag

(amino acid sequence GGGLNDIFEAQKIEWHE). GST- tagged SOS1 protein corresponding to SOS1 (amino acids 564-1049) with an amino-terminal GST-tag and a Tobacco-etch-virus (TEV) protease cleavage site was expressed in E. coli and purified by affinity chromatography on a GSH-column, followed by desalting (HiPrep 26/10 Desalting, GE Healthcare) into 20 mM Tris, 200 mM NaCl, 10% Glycerol, 1mM DTT, pH 8.0. The tag was not cleaved.

Compounds are dispensed onto assay plates (Proxiplate 384 PLUS, white, PerkinElmer) using an Access Labcyte Workstation with the Labcyte Echo 55x from a DMSO solution. For the chosen highest assay concentration of 500 µM or 100 µM (this can be changed upon request), 150 nL of compound solution are transferred from a 50 mM or 10 mM DMSO compound stock solution. Compounds are tested in duplicates. A series of 11 concentrations is transferred for each compound at which each concentration is fivefold lower than the previous one. DMSO is added such that every well has a total of 150 nL compound solution. The assay runs on a fully automated robotic system. For the assay 15 µL containing KRAS G12D protein (15 nM final assay concentration), SOS1 (10 nM final assay concentration), GTP (10 µM final assay concentration), Lance Eu-W1024 labeled Streptavidin (1.5 nM final assay concentration) and Anti-GST surelight APC (30 nM final assay concentration) mixed in assay buffer (1x PBS; 0.05% Tween20; 0.1 % BSA; filtered) are added to the 150 nl of compounds. Plates are kept at room temperature. After 60 minutes incubation time the TR-FRET signal is measured in a PerkinElmer Envision HTS Multilabel Reader using the TR-FRET LANCE Ultra specs of PerkinElmer. Each plate contains negative controls (diluted DMSO instead of test compound; described mix with KRAS G12D protein) and positive controls (diluted DMSO instead of test compound; described mix without KRAS G12D). Negative and positive control values are used for normalization.

Nucleotide Exchange assay

The ability of the compounds to affect nucleotide exchange on Ras was assessed using the method previously reported (11, 12). Briefly, the baseline fluorescence of KRAS loaded with BODIPY-GDP (Life Technologies) was recorded for 10 seconds prior to the addition of compound. A second addition of excess GTP ± SOScat was performed at 120 seconds and nucleotide exchange was monitored as a decrease in fluorescence with time. Changes in fluorescence were monitored using a Hamamatsu FDSS 6000 with readings conducted every 3 seconds for 30 minutes. Raw fluorescence data was fit to a single exponential decay function using XLfit (IDBS) software.

Synthetic procedures

List of abbreviations

AcOH	Acetic acid
MeCN	Acetonitrile
Boc	*tert*.butoxy carbonyl; di-*tert*-butyl dicarbonate
cHex	Cyclohexane
DAD	Diode array detector
DCM	Dichloromethane, CH_2Cl_2
Dppf	1,1'-Bis(diphenylphosphino)ferrocene
DIPEA	Diisopropylethyl amine

DME	1,2-Dimethoxyethane
DMF	*N,N*-Dimethylformamide
DMSO	Dimethylsulphoxide
EtOAc or EA	Ethyl acetate
EtOH	Ethanol
h	Hour(s)
Hex	Hexane
HPLC	High performance liquid chromatography
HRMS	High resolution mass spectroscopy
INT	Intermediate
KOAc	Potassium acetate
LC	Liquid Chromatography
M	Molar (mol/L)
MeOH	Methanol
µL	Microliter
µm	Micrometer
Min	Minute(s)
mL	Milliliter
Mm	Millimeter
MS	Mass spectrometry
MsCl	Methanesulfonyl chloride
Nm	Nanometer
N	Normal
NMR	Nuclear magnetic resonance
PE	Petrolether
Pd_2dba_3	Tris(dibenzylideneacetone)dipalladium(0)
$Pd(dppf)Cl_2$	[1,1′-Bis(diphenylphosphino)ferrocene]dichloropalladium(II)
Ppm	Parts per million
prot.	Protonated
RP	Reversed phase
Rt	Room temperature (20 to 25°C)
SM	Starting material
TEA	Triethylamine
TFA	Trifluoroacetic acid
THF	Tetrahydrofuran
t_R	Retention time [min]
XPhos	2-Dicyclohexylphosphino-2′,4′,6′-triisopropylbiphenyl

General Methods

If not specifically defined herein, compounds were obtained from commercially suppliers such as Sigma-Aldrich. Unless otherwise indicated all reactions were carried out in standard commercially available glassware using standard synthetic chemistry methods. Air-sensitive and moisture-sensitive reactions were performed under an atmosphere of dry nitrogen or argon with dried glassware. Commercial starting materials were used without further purification. Solvents used for reactions were of commercial "dry"- or "extra-dry" or "analytical" grade. All other solvents used were reagent grade.

Preparative RP-HPLC was carried out on Agilent or Gilson systems using columns from Waters (Sunfire C18 OBD, 5 or 10 µm, 20x50 mm, 30x50 mm or 50x150 mm; X-

Bridge C18 OBD, 5 or 10 µm, 20x50, 30x50, or 50x150 mm) or YMC (Triart C18, 5 or 10 µm, 20x50 mm, or 30x50 mm). Unless otherwise indicated compounds were eluted with MeCN/water gradients using either acidic (0.2 % HCOOH or TFA) or basic water (5 mL 2 M NH_4HCO_3 + 2 mL NH_3 (32 %) made up to 1 L with water).

NMR experiments were recorded on Bruker Avance HD 400 MHz and 500 MHz spectrometers equipped with BBO Prodigy and TCI cryoprobes at 298 K, respectively. Samples were dissolved in 600 µL DMSO-d6 and TMS was added as an internal standard. 1D 1H spectra were acquired with 30° excitation pulses and an interpulse delay of 4.2 s with 64k data points and 20 ppm sweep width.

1D 13C spectra were acquired with broadband composite pulse decoupling (WALTZ16) and an interpulse delay of 3.3 sec with 64 k data points and a sweep width of 240 ppm. Processing and analysis of 1D spectra was performed with Bruker Topspin 3.2 software. No zero filling was performed and spectra were manually integrated after automatic baseline correction. Chemical shifts are reported in ppm on the δ scale.

HSQC spectra were recorded on all samples to aid the interpretation of the data and to identify signals hidden underneath solvent peaks. Spectra were acquired with sweep widths obtained by automatic sweep width detection from 1D reference spectra in the direct dimension with 1k data points and with 210 ppm and 256 data points in the indirect dimension.

Analytical LC/MS [LC/MS(BAS1)] data were measured on an Agilent HPLC 1100 Series with Agilent LC/MSD SL detector using a Waters X-Bridge C18, 2.5 µm, 2.1x20mm column (Part.No. 186003201) and solvent A [20 mM aqueous NH_4HCO_3/ NH_3 (pH 9)] and solvent B [acetonitrile HPLC grade] as eluent (additional settings: flow 1mL/min; injection volume 5 µl; column temp. 60 °C). Standard gradient: 0.00 min:10 % B; 0.00 – 1.50 min: 10 % -> 95 % B; 1.50 – 2.00 min: 95 % B; 2.00 – 2.10 min: 95 % -> 10 % B.

HRMS data were recorded using a LTQ Orbitrap XL (Thermo Scientific) coupled with a Triversa Nanomate Nanospray ion source (ADVION Bioscience Inc.). The mass calibration was performed using the Pierce LTQ Velos ESI positive ion calibration solution from Thermo Scientific (Lot PF200011, Product Nr. 88323).

MS parameter:

Scan window	50 – 400amu, linear ion trap (LTQ) max. injection time 500ms with 1 microscan
Resolution	60000 (Orbitrap)
Mass accuracy	≤ 5ppm
Ion mode	positive
Capillary temperature	200°C
Capillary voltage	60eV
Tube lens potential	110eV

Nanomate spray-parameter for infusion mode:

NanoESI voltage	1.45kV
N_2-gas pressure	0.45psi
Sample volume	5µL

Exact mass recording:

Acquire time 0.4sec, average spectra of 10 scans
Sample dilution: 10mM DMSO stock solution diluted 1:200 in 50% MeOH +0.01% FA

Compound Syntheses

2-Formyl-4-hydroxy-benzoic acid methyl ester (27)

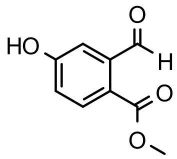

To a stirred solution of 4-hydroxy-2-methylbenzoic acid (50.0 g; 329 mmol) in MeOH (500 mL) thionyl chloride (48.9 mL; 657.3 mmol) is added under ice cooled condition and the reaction mixture is heated at 80 °C for 4 h. The solvent is evaporated and the residue is dissolved in dichloromethane (500 mL) and $NaHCO_3$ (300 mL). The organic phase is separated and the aqueous part is extracted with dichloromethane (2 x 200 mL). The organic phases are combined, washed with brine, dried over Na_2SO_4, filtered and concentrated in vacuo to yield an off white solid.

TBSCl (10.83 g; 72 mmol) is added to a stirred solution of 4-hydroxy-2-methylbenzoic acid methyl ester 24 (10.0 g; 60 mmol) in DMF (100 mL) at 0 °C. After 15 min DIPEA (22.2 mL; 120 mmol) is added to the reaction mixture and stirring is continued for overnight at room temperature. The reaction mixture is treated with water and extracted with hexane, washed with water, the combined organic phases are dried over Na_2SO_4 and concentrated under reduced pressure to obtain the crude TBS protected compound 4-(tert-Butyl-dimethyl-silanyloxy)-2-methyl-benzoic acid methyl ester 25.

N-Bromosuccinimide (361.5 g; 2031 mmol) and AIBN (74.11 g; 451.3 mmol) are added to a stirred solution of 4-(tert-Butyl-dimethyl-silanyloxy)-2-methyl-benzoic acid methyl ester 25 (126.56 g; 451.3 mmol) in benzene (3.1 L) at rt. The reaction mixture is heated to reflux overnight. The reaction mass is dissolved in DCM and filtered to remove excess NBS and then treated with hexane and filtered again. The filtrate is concentrated to get the liquid crude material 4-(tert-Butyl-dimethyl-silanyloxy)-2-dibromomethyl-benzoic acid methyl ester 26 was used directly in the next step.

Silver nitrate (52.912 g; 0.311 mol) is added to a stirred solution of 4-(tert-Butyl-dimethyl-silanyloxy)-2-dibromomethyl-benzoic acid methyl ester 26 (65.0 g; 148.33 mmol) in acetone/water (5:1) (900 mL) and the reaction mixture is stirred at room temperature for 16 h. The reaction mixture was filtered and concentrated in vacuo, taken up in EtOAc (50 mL) and is washed with water (3 x 20 mL). The organic phases are dried over Na_2SO_4, filtered and concentrated under reduced pressure. The crude brown oil is purified by column chromatography over silica gel using EtOAc/Hex (1:9, v/v) as eluent to obtain an off white solid. The solid material is then washed by 5%EtOAc\Hex to yield 2-formyl-4-hydroxy-benzoic acid methyl ester 27 (11.0 g; 61.0 mmol; 41.2 %). HPLC method: LCMSBAS1: t_{ret} [min] = 0.20; $[M-H]^+$ = 181.

5-Hydroxy-3-(2-hydroxymethyl-1H-indol-3-yl)-2,3-dihydro-isoindol-1-one (29)

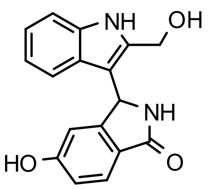

A suspension of (1H-indol-2-yl)methanol (28) (1.078 g; 6.594 mmol; commercial from ArkPharm), methyl 2-formyl-4-hydroxy-benzoate (27) (1.0 g; 5.495 mmol) and ammonia solution (28% in water, 7.816 mL; 54.952 mmol) in water (10 mL) is stirred in a capped 20 mL microwave vial and heated overnight at 85°C. The reaction mixture is diluted and dissolved with ACN, filtered and purified by preparative RP-HPLC using a ACN/water gradient as eluent to give 5-hydroxy-3-(2-hydroxymethyl-1H-indol-3-yl)-2,3-dihydro-isoindol-1-one (29) (1.048 g; 3.561 mmol; 64.8 %). HPLC method: LCMSBAS1: t_{ret} [min] = 0.46; $[M+H]^+$ = 295.

The alcohol (1 g; 340 µmol) is separated via preparative chiral SFC to give (3S)-5-hydroxy-3-[2-(hydroxymethyl)-1H-indol-3-yl]-2,3-dihydro-1H-isoindol-1-one (493 mg; 168 µmol) and (3R)-5-hydroxy-3-[2-(hydroxymethyl)-1H-indol-3-yl]-2,3-dihydro-1H-isoindol-1-one (484 mg; 164.5 µmol).

3-(6-hydroxy-3-oxo-2,3-dihydro-1H-isoindol-1-yl)-1H-indole-2-carbaldehyde (30)

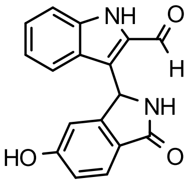

5-Hydroxy-3-[2-(hydroxymethyl)-1H-indol-3-yl]-2,3-dihydro-1H-isoindol-1-one (29) (2.0 g; 6.59 mmol) and MnO_2 (6.367 g; 65.92 mmol) are suspended in MeOH and heated to reflux for 30 min. The hot suspension is filtered and washed with hot MeOH (100 mL). After cooling, MeOH is removed under reduced pressure and the crude product is purified by flash chromatography on SiO_2 using a DCM/MeOH gradient (0%-10% MeOH). The product containing fractions are combined, concentrated under reduced pressure, dissolved in ACN/water (1:1) and freeze dried to give 3-(6-hydroxy-3-oxo-2,3-dihydro-1H-isoindol-1-yl)-1H-indole-2-carbaldehyde (30) (1.3 g; 4.45 mmol; 67.5 %). HPLC method: LCMSBAS1: t_{ret} [min] = 0.53; $[M+H]^+$ = 293.

In a similar procedure starting from the chiral separated alcohols e.g. (3S)-5-hydroxy-3-[2-(hydroxymethyl)-1H-indol-3-yl]-2,3-dihydro-1H-isoindol-1-one (156 mg; 530 µmol) the chiral aldehyde 3-[(1S)-6-hydroxy-3-oxo-2,3-dihydro-1H-isoindol-1-yl]-1H-indole-2-carbaldehyde (31S) (149 mg; 510 µmol) and aldehyde 3-[(1R)-6-hydroxy-3-oxo-2,3-dihydro-1H-isoindol-1-yl]-1H-indole-2-carbaldehyde (31R) can be synthesized.

3-{2-[(dimethylamino)methyl]-1H-indol-3-yl}-5-hydroxy-2,3-dihydro-1H-isoindol-1-one (32)

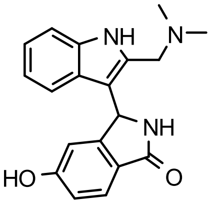

The aldehyde 3-(6-hydroxy-3-oxo-2,3-dihydro-1H-isoindol-1-yl)-1H-indole-2-carbaldehyde 30 (1.0 g; 3.42 mmol) is dissolved in DMF (10 mL) and dimethylamine (8.55 mL; 2 M solution in THF; 17.1 mmol) is added and the reaction flask is pressurized to 4 bar (58 psi) via nitrogen gas and stirred at room temperature for 15 min before sodium triacetoxyborohydride (3.63 g; 17.1 mmol) is added. The reaction mixture is stirred at room temperature overnight. The reaction mixture is filtered, the filtrate is concentrated under reduced pressure and purified by preparative RP-HPLC using a MeCN/water (5-50% MeCN, basic conditions) gradient. The product containing fractions are freeze dried to yield 3-{2-[(dimethylamino)methyl]-1H-indol-3-yl}-5-hydroxy-2,3-dihydro-1H-isoindol-1-one (32) (800 mg; 249 µmol; 72.8 %). ^{1}H NMR (400 MHz, DMSO-d_6) δ 11.05 (s, 1H), 9.99 (br s, 1H), 8.47 (s, 1H), 7.57 (d, *J*=8.36 Hz, 1H), 7.28 (d, *J*=8.11 Hz, 1H), 6.96 (ddd, *J*=1.01, 7.00, 8.20 Hz, 1H), 6.84 (dd, *J*=2.03, 8.36 Hz, 1H), 6.72 (t, *J*=7.35 Hz, 1H), 6.60 (br d, *J*=6.59 Hz, 1H), 6.52 (d, *J*=1.77 Hz, 1H), 5.93 (s, 1H), 3.72 (d, *J*=13.94 Hz, 1H), 3.58 (br d, *J*=13.18 Hz, 1H), 2.24 (s, 6H); ^{13}C NMR (100 MHz, DMSO-d_6) δ 169.7, 160.9, 151.1, 135.8, 135.1, 125.7, 124.3, 123.5, 121.0, 118.6, 118.4, 115.7, 111.3, 109.6, 109.2, 54.0, 52.3, 45.2; HRMS (CI+): calculated for $C_{19}H_{19}N_3O_2$ (MH+) 322.15500, found 322.15544, Δ 1.36 ppm; LC/MS (BAS1): $[M+H]^+$ = 322; t_R = 0.64 min.

3-{2-[(dimethylamino)methyl]-1H-indol-3-yl}-5-hydroxy-2,3-dihydro-1H-isoindol-1-one (90 mg; 280 µmol) are separated via chiral SFC to give (3S)-3-{2-[(dimethylamino)methyl]-1H-indol-3-yl}-5-hydroxy-2,3-dihydro-1H-isoindol-1-one (18) (35 mg; 109 µmol) and (3R)-3-{2-[(dimethylamino)methyl]-1H-indol-3-yl}-5-hydroxy-2,3-dihydro-1H-isoindol-1-one (33) (35 mg; 109 µmol).

18 + 33: HRMS (CI+): calculated for $C_{19}H_{19}N_3O_2$ (MH+) 322.15500, found 322.15537, Δ 1.14 ppm; HRMS (CI+): calculated for $C_{19}H_{19}N_3O_2$ (MH+) 322.15500, found 322.15570, Δ 2.16 ppm; LC/MS (BAS1): $[M+H]^+$ = 322; t_R = 0.63 min.

3-{2-[(benzylamino)methyl]-1H-indol-3-yl}-5-hydroxy-2,3-dihydro-1H-isoindol-1-one (19)

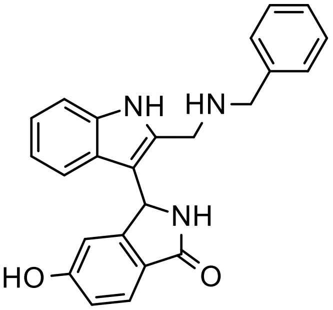

The aldehyde 3-(6-hydroxy-3-oxo-2,3-dihydro-1H-isoindol-1-yl)-1H-indole-2-carbaldehyde 30 (50.0 mg; 171 µmol) and benzylamine (20.16 mg; 188 µmol) are dissolved in DMF (0.5 mL) and stirred for 15 min at room temperature. The reaction mixture is treated with sodium triacetoxyborohydride (181.2 mg; 855 µmol) and stirring is continued for 16 h. The reaction mixture is filtered, the filtrate is concentrated under reduced pressure and purified by preparative RP-HPLC using a ACN/water (5-50% ACN) gradient. The product containing fractions are freeze dried to yield 3-{2-[(benzylamino)methyl]-1H-indol-3-yl}-5-hydroxy-2,3-dihydro-1H-isoindol-1-one (19) (51.4 mg; 134 µmol; 78.4 %). ^{1}H NMR (500 MHz, DMSO-d_6) δ 11.04 (s, 1H), 10.24 (br s, 1H), 10.01 (s, 1H), 8.47 (s, 1H), 7.57 (d, *J*=8.20 Hz, 1H), 7.36-7.40 (m, 2H), 7.28-7.35 (m, 4H), 7.19-7.27 (m, 1H), 6.96 (t, *J*=7.57 Hz, 1H), 6.85 (dd, *J*=1.89, 8.20 Hz, 1H), 6.72 (br t, *J*=7.41 Hz, 1H), 6.56 (d, *J*=1.89 Hz, 1H), 5.88 (s, 1H), 3.94 (br s, 2H), 3.73 (br d, *J*=7.57 Hz, 2H); ^{13}C NMR (125 MHz, DMSO-d_6) δ 169.6, 160.9, 151.0, 140.5, 136.8, 135.7, 128.3, 128.1, 126.8, 125.8, 124.3, 123.5, 120.9, 118.6, 118.3, 115.7, 111.3, 109.7, 108.4, 52.4, 52.2, 43.5; HRMS (CI+): calculated for $C_{24}H_{21}N_3O_2$ (MH+) 384.17065, found 384.17020, Δ -1.18 ppm; LC/MS (BAS1): $[M+H]^+$ = 384; t_R = 0.93 min.

3-{2-[(benzylamino)methyl]-1H-indol-3-yl}-5-hydroxy-2,3-dihydro-1H-isoindol-1-one (19) (15 mg; 39 µmol) was separated via chiral SFC to give (3S)-3-{2-[(benzylamino)methyl]-1H-indol-3-yl}-5-hydroxy-2,3-dihydro-1H-isoindol-1-one (34) (3 mg; 8 µmol) and (3R)-3-{2-[(benzylamino)methyl]-1H-indol-3-yl}-5-hydroxy-2,3-dihydro-1H-isoindol-1-one (35) (3 mg; 8 µmol) LC/MS (BAS1): [M+H]+ = 384; t_R = 1.00 min.

5-hydroxy-3-[2-({[(1H-indol-6-yl)methyl]amino}methyl)-1H-indol-3-yl]-2,3-dihydro-1H-isoindol-1-one (20)

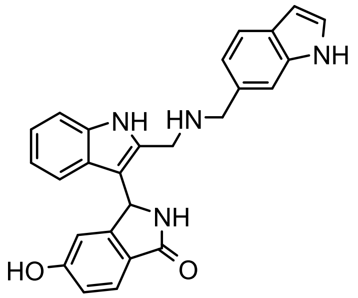

The aldehyde 3-(6-hydroxy-3-oxo-2,3-dihydro-1H-isoindol-1-yl)-1H-indole-2-carbaldehyde 30 (70.0 mg; 239 µmol) and the amine 1H-indole-6-methanamine (40.5 mg; 263 µmol) are dissolved in DMF (1 mL) and stirred for 15 min at room temperature. The reaction mixture is treated with sodium triacetoxyborohydride (253.3 mg; 1.2 mmol) and stirring is continued for 16 h. The reaction mixture is filtered, the filtrate is concentrated under reduced pressure and purified by preparative RP-HPLC using a ACN/water (5-50% ACN) gradient. The product containing fractions are freeze dried to yield 5-hydroxy-3-[2-({[(1H-indol-6-yl)methyl]amino}methyl)-1H-indol-3-yl]-2,3-dihydro-1H-isoindol-1-one (20) (68 mg; 134 µmol; 67.3 %). ^{1}H NMR (400 MHz, DMSO-d_6) δ 11.12 (s, 1H), 11.02 (br s, 1H), 10.07-10.42 (m, 1H), 8.48 (s, 1H), 8.19 (s, 1H), 7.58 (d, *J*=8.36 Hz, 1H), 7.48 (d, *J*=8.11 Hz, 1H), 7.41 (s, 1H), 7.31 (d, *J*=8.36 Hz,

1H), 7.27-7.30 (m, 1H), 7.04 (d, J=8.11 Hz, 1H), 6.94-7.00 (m, 1H), 6.85 (dd, J=2.03, 8.36 Hz, 1H), 6.72 (t, J=7.48 Hz, 1H), 6.58 (d, J=1.77 Hz, 1H), 6.50-6.56 (m, 1H), 6.38 (t, J=1.90 Hz, 1H), 5.91 (s, 1H), 4.03 (br s, 2H), 3.88 (br s, 2H); ^{13}C NMR (100 MHz, DMSO-d_6) δ 169.7, 161.0, 151.0, 136.1, 135.8, 132.3, 126.9, 125.8, 125.4, 124.3, 123.5, 121.1, 119.9, 119.8, 118.7, 118.5, 115.8, 111.4, 111.2, 109.8, 108.8, 101.0, 52.9, 52.3, 43.3;1 carbon not detectable; HRMS (CI+): calculated for $C_{26}H_{22}N_4O_2$ (MH+) 423.18155, found 423.18060, Δ -2.25 ppm; LC/MS (BAS1): $[M+H]^+$ = 423; t_R = 0.93 min.

5-Hydroxy-3-[2-({[(1H-indol-6-yl)methyl]amino}methyl)-1H-indol-3-yl]-2,3-dihydro-1H-isoindol-1-one (20) (60.0 mg; 142 µmol) was separated via chiral SFC to give (3S)-5-hydroxy-3-[2-({[(1H-indol-6-yl)methyl]amino}methyl)-1H-indol-3-yl]-2,3-dihydro-1H-isoindol-1-one (36) (16 mg; 38 µmol) and (3R)-5-hydroxy-3-[2-({[(1H-indol-6-yl)methyl]amino}methyl)-1H-indol-3-yl]-2,3-dihydro-1H-isoindol-1-one (37) (17 mg; 40 µmol). LC/MS (BAS1): $[M+H]^+$ = 423; t_R = 0.93 min.

5-hydroxy-3-[2-({[(1-methyl-1H-indol-6-yl)methyl]amino}methyl)-1H-indol-3-yl]-2,3-dihydro-1H-isoindol-1-one (21)

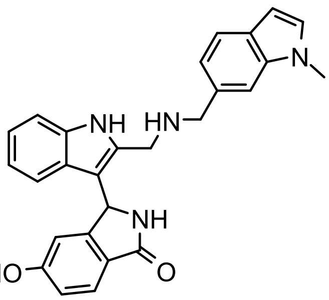

The aldehyde 3-(6-hydroxy-3-oxo-2,3-dihydro-1H-isoindol-1-yl)-1H-indole-2-carbaldehyde 30 (50.0 mg; 96.9%; 166 µmol) and (1-methyl-1H-indole-6-yl)-methylamine (33.6 mg; 199 µmol) are dissolved in DMF (1 mL) and stirred for 15 min at room temperature. The reaction mixture is treated with sodium triacetoxyborohydride (175.9 mg; 830 µmol) and stirring is continued for 16 h. The reaction mixture is filtered, the filtrate is concentrated under reduced pressure and purified by preparative RP-HPLC using a ACN/water (5-50% ACN) gradient. The product containing fractions are freeze dried to yield 5-hydroxy-3-[2-({[(1-methyl-1H-indol-6-yl)methyl]amino}methyl)-1H-indol-3-yl]-2,3-dihydro-1H-isoindol-1-one (21) (52 mg; 119 µmol; 71.8 %). ^{1}H NMR (500 MHz, DMSO-d_6) δ 11.11 (s, 1H), 10.15-10.37 (m, 1H), 8.49 (s, 1H), 8.20 (s, 1H), 7.58 (d, J=8.20 Hz, 1H), 7.48 (d, J=7.88 Hz, 1H), 7.44 (s, 1H), 7.32 (d, J=7.88 Hz, 1H), 7.25 (d, J=3.15 Hz, 1H), 7.07 (d, J=8.20 Hz, 1H), 6.97 (t, J=7.60 Hz, 1H), 6.85 (dd, J=2.21, 8.20 Hz, 1H), 6.73 (br t, J=7.41 Hz, 1H), 6.60 (d, J=1.58 Hz, 1H), 6.57 (br dd, J=0.79, 2.99 Hz, 1H), 6.36 (d, J=3.15 Hz, 1H), 5.89 (s, 1H), 4.00 (br s, 2H), 3.86-3.92 (m, 2H), 3.72 (s, 3H); ^{13}C NMR (125 MHz, DMSO) δ 169.6, 161.0, 151.0, 136.6, 136.5, 135.8, 132.9, 129.6, 127.2, 125.8, 124.3, 123.5, 121.1, 120.1, 119.9, 118.7, 118.4, 115.8, 111.4, 109.8, 109.2, 108.6, 100.2, 52.9, 52.3, 43.2, 32.5; HRMS (CI+): calculated for $C_{27}H_{24}N_4O_2$ (MH+) 437.19720, found 437.19725, Δ 0.11 ppm; LC/MS (BAS1): $[M+H]^+$ = 437; t_R = 1.00 min.

The pure enantiomers are synthesized analogously starting from the chiral aldehydes 31S and 31R to yield (S)-5-Hydroxy-3-(2-{[(1-methyl-1H-indol-6-ylmethyl)-amino]-methyl}-1H-indol-3-yl)-2,3-dihydro-isoindol-1-one (38) and (R)-5-Hydroxy-3-(2-{[(1-methyl-1H-indol-6-ylmethyl)-amino]-methyl}-1H-indol-3-yl)-2,3-dihydro-isoindol-1-one (39).

38 +39: HRMS (CI+): calculated for $C_{27}H_{24}N_4O_2$ (MH+) 437.19720, found 437.19742, Δ 0.50 ppm; HRMS (CI+): calculated for $C_{27}H_{24}N_4O_2$ (MH+) 437.19720, found 437.19719, Δ - 0.03 ppm; LC/MS (BAS1): $[M+H]^+$ = 437; t_R = 1.07 min.

3-[2-({[(1-benzyl-1H-indol-6-yl)methyl]amino}methyl)-1H-indol-3-yl]-5-hydroxy-2,3-dihydro-1H-isoindol-1-one (22)

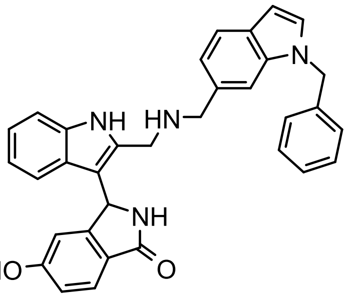

In a glass vial aldehyde 3-(6-hydroxy-3-oxo-2,3-dihydro-1H-isoindol-1-yl)-1H-indole-2-carbaldehyde (30) (12 mg; 96.9%; 40 µmol) is dissolved in DMF (0.5 mL) and (1-benzyl-1H-indol-6-yl)methanamine (10 mg; 42 µmol) is added and stirred at room temperature for 15 min. Sodium triacetoxyborohydride (42.2 mg; 200 µmol) is added and the mixture is stirred at room temperature for 2 h. Sodium triacetoxyborohydride (42.2 mg; 200 µmol) is added again, and the reaction mixture is stirred at room temperature overnight. The reaction mixture is diluted by addition of ACN/H_2O (1:1; 200 µL), filtered through a syringe filter and purified by prep. HPLC (Gilson; column: SunFire Prep C18, 5µm (30*50), gradient, acidic conditions ACN/water (5:95 to 60:40 in 8 min. flowrate: 50 mL/min wavelength: 222 nm). The product containing fraction is freeze-dried to yield 3-[2-({[(1-benzyl-1H-indol-6-yl)methyl]amino}methyl)-1H-indol-3-yl]-5-hydroxy-2,3-dihydro-1H-isoindol-1-one (22) (6 mg; 12 µmol; 29.4 %). ^{1}H NMR (500 MHz, DMSO-d_6) δ 11.04 (s, 1H), 9.86-10.21 (m, 1H), 8.48 (s, 1H), 7.58 (d, *J*=8.36 Hz, 1H), 7.49 (d, *J*=8.11 Hz, 1H), 7.45 (s, 1H), 7.41 (d, *J*=3.29 Hz, 1H), 7.30 (d, *J*=8.11 Hz, 1H), 7.16-7.27 (m, 4H), 7.11 (d, *J*=6.60 Hz, 2H), 7.07 (d, *J*=7.60 Hz, 1H), 6.96 (ddd, *J*=0.90, 7.03, 8.17 Hz, 1H), 6.85 (dd, *J*=2.03, 8.11 Hz, 1H), 6.72 (t, *J*=7.48 Hz, 1H), 6.58 (d, *J*=1.77 Hz, 1H), 6.49-6.56 (m, 1H), 6.44 (d, *J*=3.04 Hz, 1H), 5.86 (s, 1H), 5.35 (s, 2H), 3.93 (s, 2H), 3.81 (br s, 2H); ^{13}C NMR (125 MHz, DMSO-d_6) δ 169.7, 161.0, 151.1, 138.5, 137.1, 136.1, 135.7, 133.8, 129.0, 128.6, 127.4, 127.3, 127.0, 125.9, 124.3, 123.6, 121.0, 120.3, 120.0, 118.6, 118.4, 115.8, 111.3, 109.8, 109.3, 108.3, 101.1, 53.1, 52.3, 49.0, 43.5; HRMS (CI+): calculated for $C_{33}H_{28}N_4O_2$ (MH+) 513.22850, found 513.22817, Δ - 0.65 ppm; LC/MS (BAS1): $[M+H]^+$ = 513; t_R = 1.24 min.

The pure enantiomers are synthesized analogously starting from the chiral aldehydes 31S and 31R to yield (3S)-3-[2-({[(1-benzyl-1H-indol-6-ylmethyl)-amino]-methyl}-1H-indol-3-yl)-5-hydroxy-2,3-dihydro-1H-isoindol-1-one (40) and (3R)-3-[2-({[(1-benzyl-

1H-indol-6-ylmethyl)-amino]-methyl}-1H-indol-3-yl)-5-hydroxy-2,3-dihydro-1H-isoindol-1-one (41)

40+ 41: HRMS (CI+): calculated for $C_{33}H_{28}N_4O_2$ (MH+) 513.22850, found 513.22828, Δ - 0.43 ppm; HRMS (CI+): calculated for $C_{33}H_{28}N_4O_2$ (MH+) 513.22850, found 513.22890, Δ 0.77 ppm; LC/MS (BAS1): $[M+H]^+ = 513$; $t_R = 1.24$ min.

1-(1-Methyl-1H-imidazol-4-ylmethyl)-1H-indole-6-carbonitrile (42)

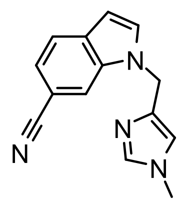

In a 50 mL round-buttom flask 6-cyanoindole (200 mg; 1.337 mmol) is dissolved in 1,3-dimethyl-3,4,5,6-tetrahydro-2(1H)-pyrimidinone (2.0 mL; 16.54 mmol), cooled in an ice-bath and treated with sodium hydride (55%; 128.3 mg; 2.94 mmol) and stirred at room temperature for additional 2 h. Tetrabutylammonium iodide (TBAI, 49.37 mg; 0.134 mmol) and 4-(chloromethyl)-1-methyl-1H-imidazole (261.78 mg; 2.005 mmol) are added to the reaction mixture and stirred overnight at room temperature. Reaction control showed incomplete 6-cyanoindole consumption and therefore an additional equivalent sodium hydride and 4-(chloromethyl)-1-methyl-1H-imidazole are added again in two portions and stirred for additional 32 h. The reaction mixture is quenched by adding MeOH (2 mL), diluted with water and extracted with EA. The combined organic layer is dried over $MgSO_4$, filtered and evaporated. The crude product is purified by preparative RP-HPLC using an ACN/water gradient (5-50% ACN) as eluent to yield 1-(1-methyl-1H-imidazol-4-ylmethyl)-1H-indole-6-carbonitrile (42) (209 mg; 885 µmol; 66.2 %). LC/MS (BAS1): $[M+H]^+ = 237$; $t_R = 0.99$ min.

C-[1-(1-Methyl-1H-imidazol-4-ylmethyl)-1H-indol-6-yl]-methylamine (43)

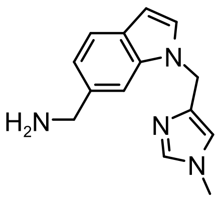

42 (95 mg; 402 µmol) is dissolved in ammonia in MeOH (7N, 3 mL) and hydrogenated over Raney-Ni (4 mg; 40 µmol) under pressure overnight. After complete consumption of the starting material the reaction mixture is filtrated, concentrated under vacuo and purified by preparative RP-HPLC using a MeCN/water (5-50% ACN) gradient under basic conditions. The product containing fractions are freeze dried to yield unstable C-[1-(1-Methyl-1H-imidazol-4-ylmethyl)-1H-indol-6-yl]-methylamine (43) (56 mg; 233 µmol; 58 %), which was immediately used for the next reaction step.

5-Hydroxy-3-[2-({[1-(1-methyl-1H-imidazol-4-ylmethyl)-1H-indol-6-ylmethyl]-amino}-methyl)-1H-indol-3-yl]-2,3-dihydro-isoindol-1-one (23)

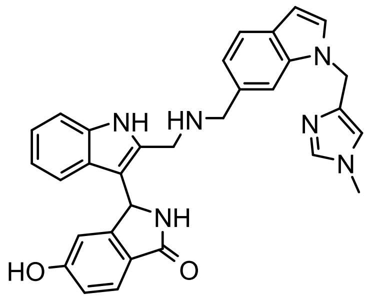

In a glass vial 3-(6-hydroxy-3-oxo-isoindolin-1-yl)-1H-indole-2-carbaldehyde (55.0 mg; 179 µmol) (30) is dissolved in DMF (1.0 ml) and C-[1-(1-methyl-1H-imidazol-4-ylmethyl)-1H-indol-6-yl]-methylamine (49.4 mg; 206 µmol) (43) is added. The reaction mixture is stirred at room temperature for 15 min before sodium triacetoxyborohydride (190 mg; 895 µmol) is added and the mixture is stirred at room temperature overnight. The reaction mixture is diluted with ACN/water 1:1 (200 µL), filtered through a syringe filter and purified by preparative RP-HPLC (4B_0550_POS) using an ACN/water gradient under acidic conditions. The product containing fractions are freeze dried to give 5-hydroxy-3-[2-({[1-(1-methyl-1H-imidazol-4-ylmethyl)-1H-indol-6-ylmethyl]-amino}-methyl)-1H-indol-3-yl]-2,3-dihydro-isoindol-1-one (23) (58 mg; 112 µmol; 62.8 %). ^{1}H (500 MHz, DMSO-d_6) δ 11.15 (s, 1H), 10.09-10.37 (m, 1H), 8.49 (s, 1H), 7.52-7.63 (m, 2H), 7.43-7.51 (m, 2H), 7.26-7.35 (m, 2H), 7.05 (br d, *J*=8.20 Hz, 1H), 6.97 (ddd, *J*=0.95, 7.09, 8.04 Hz, 1H), 6.94 (s, 1H), 6.85 (dd, *J*=1.89, 8.20 Hz, 1H), 6.73 (br t, *J*=7.41 Hz, 1H), 6.60 (d, *J*=1.89 Hz, 1H), 6.52-6.58 (m, 1H), 6.36 (d, *J*=3.15 Hz, 1H), 5.92 (s, 1H), 5.14 (s, 2H), 4.01 (br s, 2H), 3.88 (br s, 2H) 3H under water; ^{13}C NMR (125 MHz, DMSO-d_6) δ 169.7, 161.0, 151.1, 138.0, 137.9, 136.4, 135.8, 135.8, 132.7, 128.7, 127.3, 125.8, 124.3, 123.5, 121.1, 120.2, 119.9, 118.7, 118.4, 115.8, 111.4, 109.8, 109.6, 108.7, 100.5, 53.0, 52.3, 43.3, 43.2, 32.9; 1 carbon not visible; HRMS (CI+): calculated for $C_{31}H_{28}N_6O_2$ (MH+) 517.23465, found 517.23460, Δ - 0.10 ppm; LC/MS (BAS1): $[M+H]^+$ = 517; t_R = 1.03 min.

5-Hydroxy-3-[2-({[1-(1-methyl-1H-imidazol-4-ylmethyl)-1H-indol-6-ylmethyl]-amino}-methyl)-1H-indol-3-yl]-2,3-dihydro-isoindol-1-one (23) (45 mg; 87 µmol) is separated via chiral SFC to give BI-2852 (1) (S)-5-Hydroxy-3-[2-({[1-(1-methyl-1H-imidazol-4-ylmethyl)-1H-indol-6-ylmethyl]-amino}-methyl)-1H-indol-3-yl]-2,3-dihydro-isoindol-1-one (13 mg; 25 µmol; > 98% ee) and BI-2853 (44) (R)-5-Hydroxy-3-[2-({[1-(1-methyl-1H-imidazol-4-ylmethyl)-1H-indol-6-ylmethyl]-amino}-methyl)-1H-indol-3-yl]-2,3-dihydro-isoindol-1-one (15 mg; 29 µmol; only 73% ee due to peak tailing).

Enantiomeric pure 44 was also produced starting from the enantiomeric pure aldehyde 3-((R)-6-hydroxy-3-oxo-2,3-dihydro-1H-isoindol-1-yl)-1H-indole-2-carbaldehyde 31R (30.4 mg; 104 µmol) via reductive amination with C-[1-(1-Methyl-1H-imidazol-4-ylmethyl)-1H-indol-6-yl]-methylamine (25 mg; 104 µmol) (43) giving (R)-5-Hydroxy-3-[2-({[1-(1-methyl-1H-imidazol-4-ylmethyl)-1H-indol-6-ylmethyl]-amino}-methyl)-1H-indol-3-yl]-2,3-dihydro-isoindol-1-one (44)(14 mg; 27 µmol; 26.1 %).

1+44: HRMS (CI+): calculated for $C_{31}H_{28}N_6O_2$ (MH+) 517.23465, found 517.23525, Δ 1.16 ppm; HRMS (CI+): calculated for $C_{31}H_{28}N_6O_2$ (MH+) 517.23465, found 517.23497, Δ 0.62 ppm; LC/MS (BAS1): $[M+H]^+$ = 517; t_R = 1.03 min.

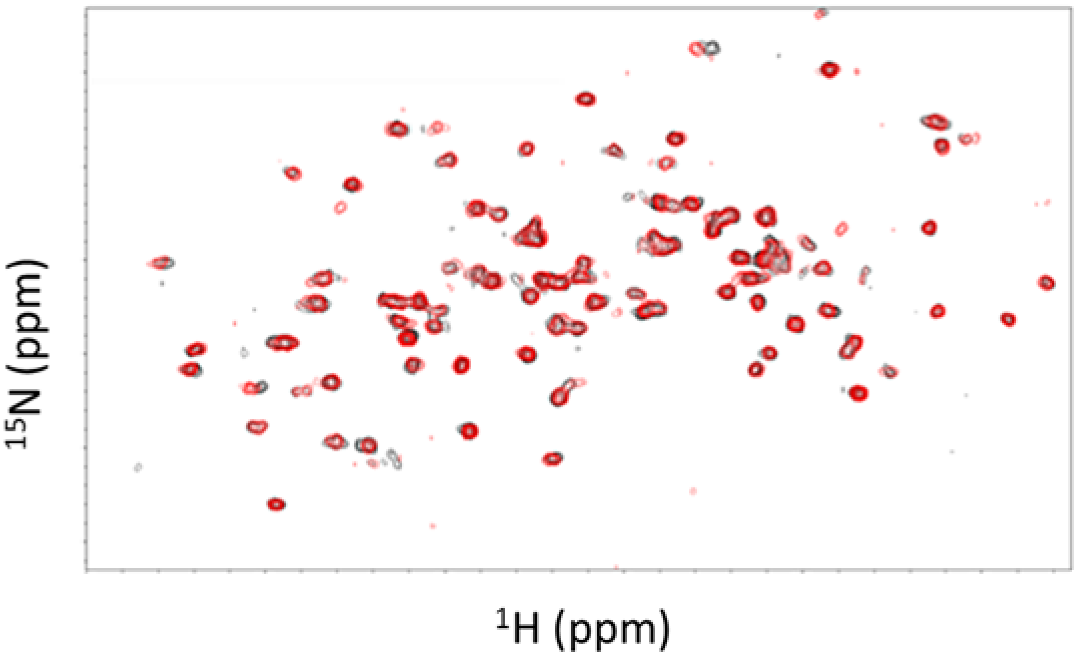

Figure S1. NMR Spectra of Fragment Hit. Superimpositions of spectra of 50 µM uniformely ^{15}N labeled GCP-KRAS^{G12D} in the absence (black) and presence of 500 µM (2) (red). Assignments of cross peaks experiencing chemical shift perturbations were taken from (Maurer et al. 2012).

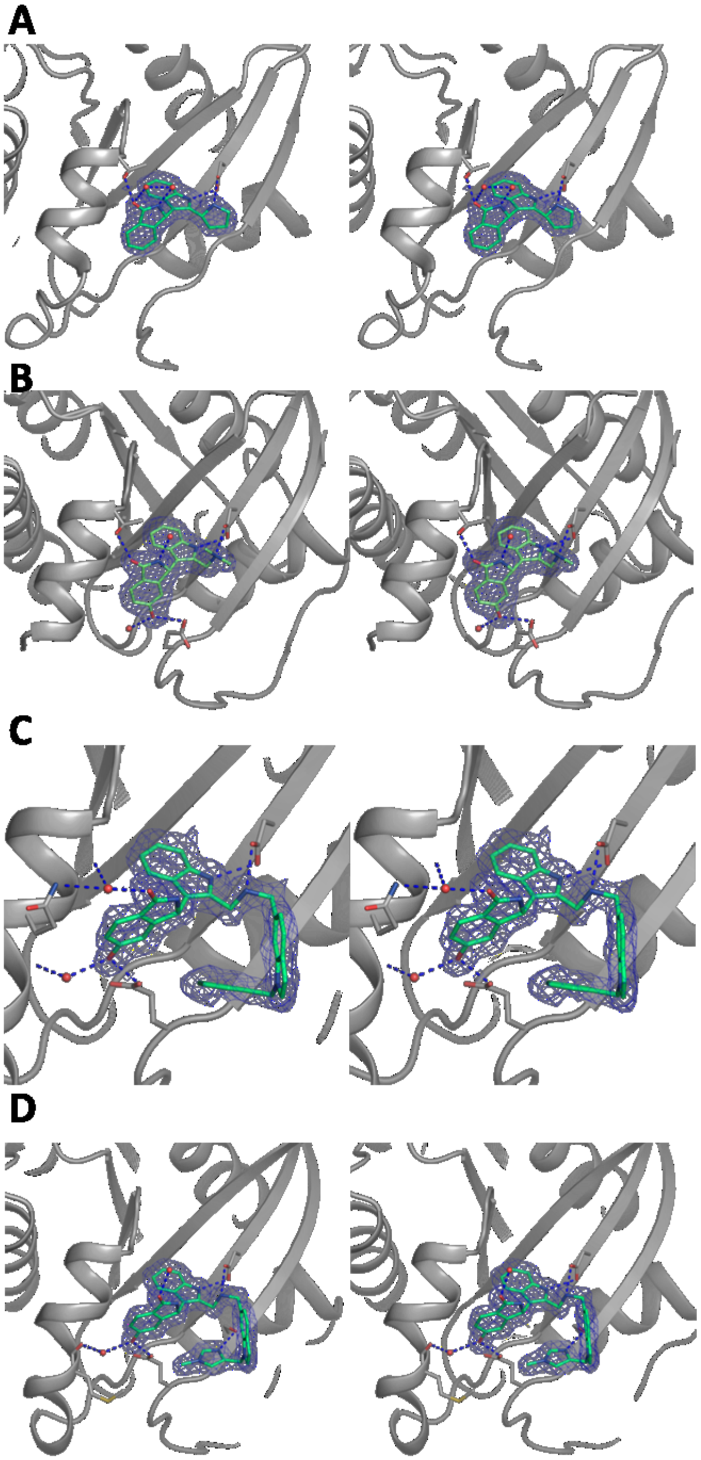
A
B
C
D

Figure S2. Stereo image of co-crystallized ligands bound to KRAS-G12D (GCP) (wall-eye stereo). The refined $2F_o$-F_c electron density is contoured at 1 σ showing the SII pocket with A) **15**, B) **18** , C) **22** and D) **BI 2852**, each with electron density around ligand.

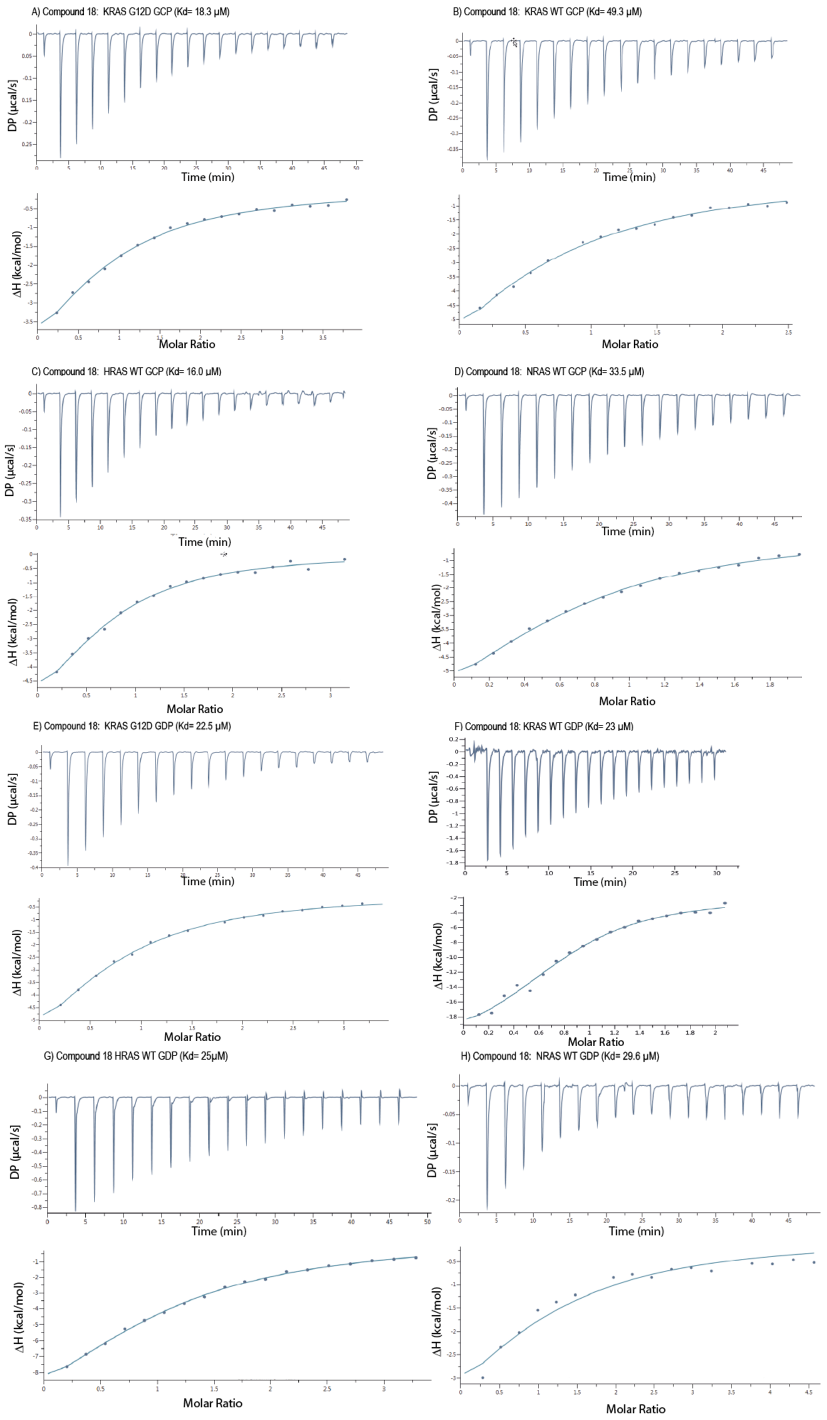

Figure S3. Representatitive ITC dose response curves for 18 (all measurements were repeated at least twice). A) ITC curve for **18** binding to GCP-$KRAS^{G12D}$. B) ITC curve for **18** binding to GCP-$KRAS^{wt}$ C) ITC curve for **18** binding to GCP-$HRAS^{wt}$ D) ITC curve for **18** binding to GCP-$NRAS^{wt}$ E) ITC curve for **18** binding to GDP-$KRAS^{G12D}$ F) ITC curve for **18** binding to GDP-$KRAS^{wt}$ G) ITC curve for **18** binding to GDP-$HRAS^{wt}$ H) ITC curve for **18** binding to GDP-$NRAS^{wt}$.

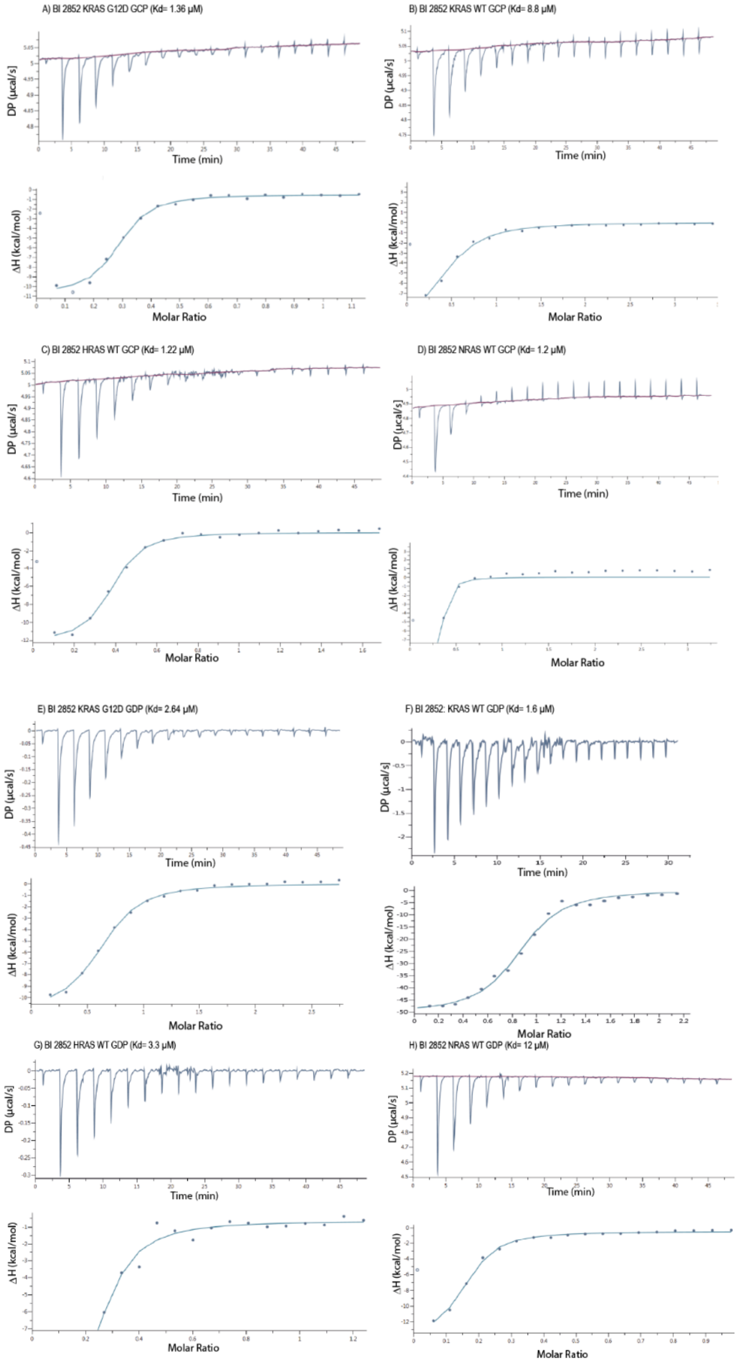

Figure S4. Representative ITC dose response curves for BI-2852 (all measurements were repeated at least twice). A) ITC curve for **BI-2852** binding to GCP-KRAS^{G12D}. B) ITC curve for **BI-2852** binding to GCP-KRAS^{wt} C) ITC curve for **BI-2852** binding to GCP-HRAS^{wt} D) ITC curve for **BI-2852** binding to GCP-NRAS^{wt} E) ITC curve for **BI-2852** binding to GDP-KRAS^{G12D} F) ITC curve for **BI-2852** binding to GDP-KRAS^{wt} G) ITC curve for **BI-2852** binding to GDP-HRAS^{wt} H) ITC curve for **BI-2852** binding to GDP-NRAS^{wt}.

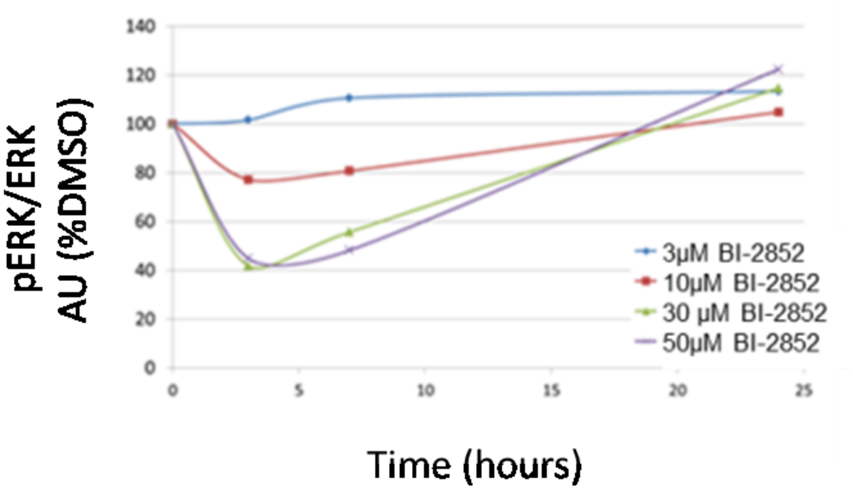

Figure S5. Time course MSD analysis of pERK levels in NCI-H358 cells after treatment of BI-2852.

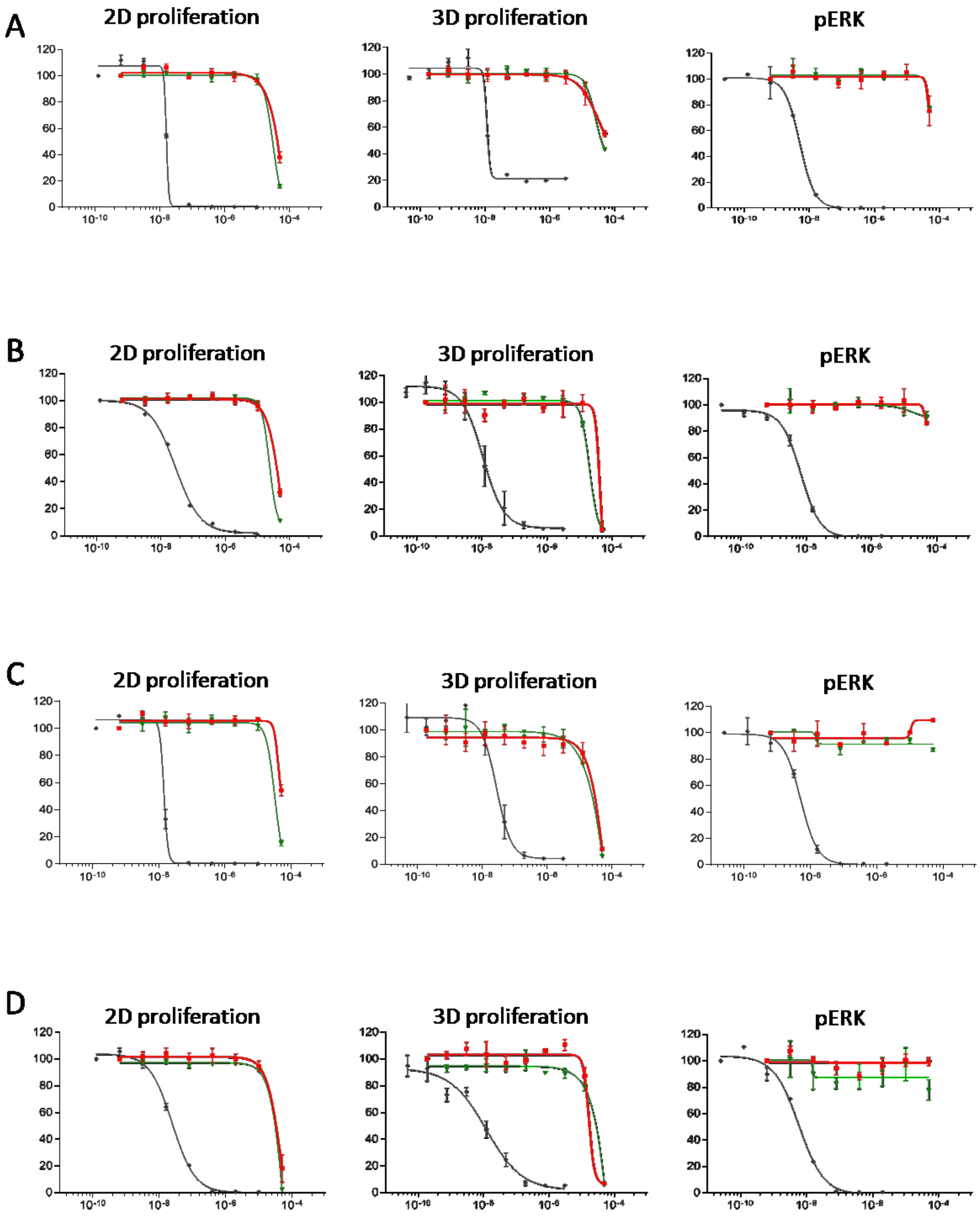

Figure S6. Effect of BI 2852 on BRAF V600E driven cell lines. A375 (A), RKO (B), Colo201 (C) and SK-Mel28 (D) cells were tested in three different assays (2D proliferation, 3D proliferation and pERK modulation) in the presence of BI 2852 (red line), compound 44 (green line) and control compounds (black line, Panobinostat in proliferation assays, Trametinib in pERK assay). Error bars indicate standard deviations of duplicates or triplicates (All these experiments have been performed once but as the picture was consistent for all 4 cell lines no further repetition was seen as necessary).

Table S1. GCP-KRAS NMR K_D measurements for 2-Methylaminoindole fragments.

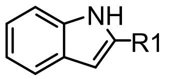

**Compound**	**R1**	**K_D T35S GCP [µM]**
**8**	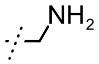	1300
**9**	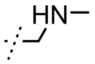	1700
**10**	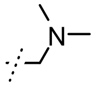	2600
**11**	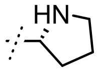	850
**12**	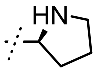	820
**13**	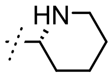	1900
**14**	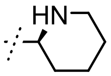	1200

Table S2. GCP-KRAS NMR K_D measurements for Dihydroisoindolinone containing indoles.

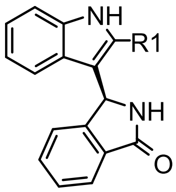

Compound	R1	K_D T35S GCP [µM]	K_D T35 GCP [µM]
**15**	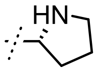	440	460
**16**	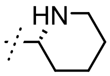	340	170
**17**	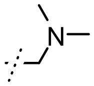	n.d.	110*

*data is for the racemic mixture

Table S3. NMR and ITC K_D measurements for 18.

Biophysical Assay	K_D [μM]	Number of repeats	Standard deviation
K_D(HSQC) GCP-KRAS^{G12D}	17	1	6.0*
K_D(HSQC) GDP-KRAS^{G12D}	16	1	2.0*
K_D(ITC) GCP-KRAS^{G12D}	22	2	3.4
K_D(ITC) GCP-KRAS^{wt}	49	1	--
K_D(ITC) GCP-HRAS^{wt}	6.7	2	0.5
K_D(ITC) GCP-NRAS^{wt}	33	1	--
K_D(ITC) GDP-KRAS^{G12D}	29	2	6.1
K_D(ITC) GDP-KRAS^{wt}	15	2	7.9
K_D(ITC) GDP-HRAS^{wt}	54	2	5.5
K_D(ITC) GDP-NRAS^{wt}	33	2	3.0

* Standard deviation for NMR data was obtained from a single K_D titration with three different peaks fitted independently.

Table S4. Biochemical activity of pendant aminomethyl derivatives.

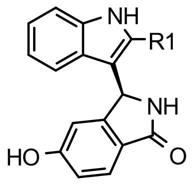

Compound	R1	IC_{50}(FRET) GTP-$KRAS^{G12D}$::SOS1 [μM]*
**18**	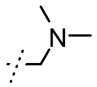	33
**19**	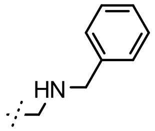	29
**20**	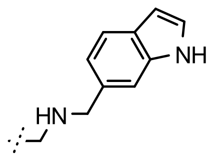	7.5
**21**	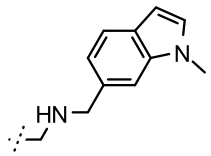	5.7
**22**	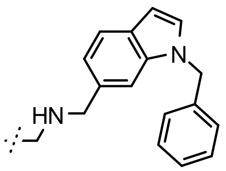	0.87

*All FRET data was performed in duplicate

Table S5. ITC K_D measurements for BI-2852 with GDP and GCP RAS isoforms.

ITC Assay	K_D [µM]	Number of repeats	Standard deviation
K_D(ITC) GCP-KRAS^{G12D}	0.74	3	0.4
K_D(ITC) GCP-KRAS^{wt}	7.5	2	1.4
K_D(ITC) GCP-HRAS^{wt}	0.57	2	0.1
K_D(ITC) GCP-NRAS^{wt}	1.3	2	0.1
K_D(ITC) GDP-KRAS^{G12D}	2.0	2	0.7
K_D(ITC) GDP-KRAS^{wt}	1.1	2	0.5
K_D(ITC) GDP-HRAS^{wt}	2.5	2	0.6
K_D(ITC) GDP-NRAS^{wt}	8.3	3	2.7

Table S6. Biochemical Data for BI-2852, 44 and ARS-1620.

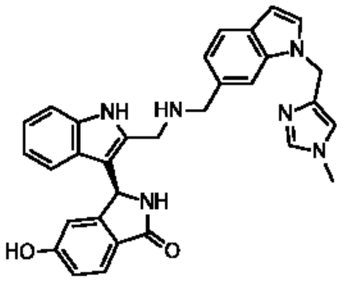 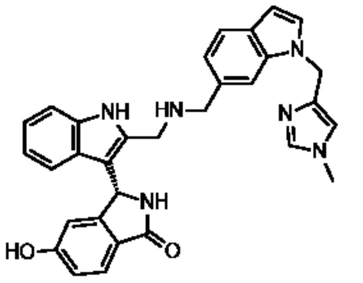 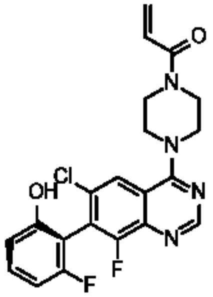

Biochemical Assay	BI-2852 [nM]	44 [nM]	ARS 1620 [nM]
IC_{50} GDP-KRAS^{G12C}::SOS1	450	3300	590
IC_{50} GTP-KRAS^{G12C}::SOS1	360	1900	>10000
IC_{50} GDP-KRAS^{G12D}::SOS1	260	2500	>10000
IC_{50} GTP-KRAS^{G12D}::SOS1	490	4400	>10000
IC_{50} GTP-KRAS^{G12C}::CRAF	180	590	>10000
IC_{50} GTP-KRAS^{G12C}::PI3Kα	100	580	>10000
IC_{50} GTP-KRAS^{G12D}::CRAF	770	ND	ND
IC_{50} GTP-KRAS^{G12D}::PI3Kα	500	ND	ND
IC_{50} GTP-KRAS^{wt} ::SOS1	490	ND	ND
IC_{50} GTP-KRAS^{wt} ::CRAF	740	ND	ND
IC_{50} GTP-KRAS^{wt} ::PI3Kα	250	ND	ND

Measurements of the protein-protein interactions of GTP-KRAS and SOS1, CRAF and PI3Kα and GDP-KRAS and SOS1 including mutant and wildtype KRAS. Assay format = luminescent oxygen channeling immunoassay. ND = not determined. All measurements are performed in duplicate.

Table S7. Crystallographic data collection and refinement statistics (MR)

	6GJ5 (15)	6GJ6 (18)	6GJ7 (22)	6GJ8 (BI 2852)
**Data collection**				
**Resolution Limit defined by**	XDS	STARANISO	STARANISO	STARANISO
Space group	$P\ 1\ 2_1\ 1$	P 63	C 2 2 21	C 2 2 21
Cell dimensions				
a, *b*, *c* (Å)	42.07, 40.04, 94.28	85.9, 85.9, 47.5	41.8, 116.67, 91.8	41.6, 116.84, 91.48
α, β, γ (°)	90.0, 101.14, 90.0	90, 90, 120	90, 90, 90	90, 90, 90
Resolution (Å)	1.499	1.761 (1.902) [a]	1.671 (1.902)[a]	1.66 (1.7)[a]
R_{merge}	7.5	6.6	18.6	3.9
$I / \sigma I$	2.7	21.2	9.9	31.9
Completeness (%)	99.64	93.27 (100.0)[b]	78.13 (100.0)[b]	85.9 (100.0)[b]
Redundancy	4.1	9.5	6.4	5.8
**Refinement**				
Resolution (Å)	1.499	1.8	1.671	1.66
No. reflections	49619	18617	20772	23023
R_{work} / R_{free}	18.43/20.29	18.14/20.97	22.7/25.7	17.38/20.11
No. atoms				
Protein	2651	1297	1325	1382
Ligands	114	61	72	72
Water	358	142	202	234
B-factors				
Protein	30.45	40.37	19.25	13.79
Ligand/ion	23.52	29.49	11.74	8.85
Water	40.15	52.73	28.11	27.71
R.m.s. deviations				
Bond lengths(Å)/ angles) (°)	0.005/1.05	0.012/1.51	0.008/1.01	0.013/1.52

[a] approximate effective resolution in parentheses
b resulting completeness after STARANISO for spherical / elliptical shells

References

1. Jerabek-Willemsen M, Wienken CJ, Braun D, Baaske P, & Duhr S (2011) Molecular interaction studies using microscale thermophoresis. *Assay Drug Dev Technol* 9(4):342-353.
2. Mayer M & Meyer B (1999) Characterization of Ligand Binding by Saturation Transfer Difference NMR Spectroscopy. *Angew Chem Int Ed Engl* 38(12):1784-1788.
3. Ross A & Senn H (2003) Automation of biomolecular NMR screening. *Curr Top Med Chem* 3(1):55-67.
4. Pervushin KV, Wider G, & Wuthrich K (1998) Single Transition-to-single Transition Polarization Transfer (ST2-PT) in [15N,1H]-TROSY. *J Biomol NMR* 12(2):345-348.
5. Eberth A & Ahmadian MR (2009) In vitro GEF and GAP assays. *Curr Protoc Cell Biol* Chapter 14:Unit 14 19.
6. Vonrhein C*, et al.* (2011) Data processing and analysis with the autoPROC toolbox. *Acta Crystallogr D Biol Crystallogr* 67(Pt 4):293-302.
7. Collaborative Computational Project N (1994) The CCP4 suite: programs for protein crystallography. *Acta Crystallogr D Biol Crystallogr* 50(Pt 5):760-763.
8. Emsley P, Lohkamp B, Scott WG, & Cowtan K (2010) Features and development of Coot. *Acta Crystallogr D Biol Crystallogr* 66(Pt 4):486-501.
9. Smart O*, et al.* (2011) Grade (http://www.globalphasing.com). Global Phasing Ltd., Cambridge. *United Kingdom*.
10. Chen VB*, et al.* (2010) MolProbity: all-atom structure validation for macromolecular crystallography. *Acta Crystallogr D Biol Crystallogr* 66(Pt 1):12-21.
11. Sun Q*, et al.* (2012) Discovery of small molecules that bind to K-Ras and inhibit Sos-mediated activation. *Angew Chem Int Ed Engl* 51(25):6140-6143.
12. Burns MC*, et al.* (2014) Approach for targeting Ras with small molecules that activate SOS-mediated nucleotide exchange. *Proceedings of the National Academy of Sciences*:201315798.

Author Contributions

D.B.M., D.K, A.M, M.G, L.J.M wrote the manuscript with input from all authors. S.W.F., A.W, M.P, edited the manuscript. A.M. wrote the synthesis supporting information. D.K. supervised the FBS screening campaigns. A.Z. supervised protein production, ITC experiments and KD determination. G.S. performed protein expression and production, nucleotide exchange. J.S. and Q.S. performed SOS1 catalyzed nucleotide exchange assays. M.M and M.Z. performed the NMR experiments and M.Z. supervised the DSF, MST and NMR measurements of the FBS screening. F.M. performed MST measurements. C.G. and Q.S performed protein preparation and NMR-based fragment screening. D.K., A.Z., B.W. and J.H. were responsible for all ITC experiments. B.W., J.P. and D.K. designed and performed the crystallization experiments. L.J.M and A.M. supervised the chemistry team. L.J.M, A.M., A.G. and A.W. designed molecules and synthetic strategies. D.C., P.G., J.K.-Ö., P.K., S.K., C.P. and Y.S. synthesized the compounds and developed chemistry routes. R.K. performed analytics of compounds synthesized. W.H. performed chiral separation and chiral analysis. C.S. measured HRMS Data to obtain the exact mass for all synthesized compounds. M.G. and M.K. designed biological experiments and supervised the biology team. T.Ge. designed and supervised the biochemical Alpha assays. T.Gm, D.H., S.F., J.S., K.S.and B.S. established and performed biochemical assays. R.S., S.M.-M., L.L. and A.S. established and performed cellular assays. J.R. supervised the DMPK team. M.P. was responsible for the biology strategy. D.B.M. and S.W.F. were responsible for the medicinal chemistry strategy.
